# Internal Training Load Perceived by Athletes and Planned by Coaches: A Systematic Review and Meta-Analysis

**DOI:** 10.1186/s40798-022-00420-3

**Published:** 2022-03-04

**Authors:** Allan Inoue, Priscila dos Santos Bunn, Everton Crivoi do Carmo, Eduardo Lattari, Elirez Bezerra da Silva

**Affiliations:** 1 Exercise and Sport Sciences Postgraduate Program, Rio de Janeiro State University, Rio de Janeiro, Brazil; 2 Research Group on Exercise and Health Science, Rio de Janeiro State University, Rio de Janeiro, Brazil; 3 Exercise Sciences Research Laboratory, Physical Education Center Admiral Adalberto Nunes (CEFAN), Brazilian Navy, Rio de Janeiro, Brazil; 4 Department of Physical Education, Senac University Center, São Paulo, Brazil; 5 Physical Activity Sciences Postgraduate Program, Salgado de Oliveira University, Niterói, Brazil

**Keywords:** Internal training load, External training load, Rating of perceived exertion, Session rating of perceived exertion

## Abstract

**Background:**

Rating of perceived exertion (RPE) and session RPE (sRPE) has been widely used to verify the internal load in athletes. Understanding the agreement between the training load prescribed by coaches and that perceived by athletes is a topic of great interest in sport science.

**Objective:**

This systematic review and meta-analysis aimed to investigate differences between the training/competition load perceived by athletes and prescribed/intended/observed by coaches.

**Methods:**

A literature search (September 2020 and updated in November 2021) was conducted using PubMed, Web of Science, Embase, and SPORTDiscus databases. The protocol was registered in the Open Science Framework (osf.io/wna4x). Studies should include athletes and coaches of any sex, age, or level of experience. The studies should present outcomes related to the RPE or sRPE for any scale considering overall training/competition sessions (physical, strength, tactical, technical, games) and/or classified into three effort categories: easy, moderate, and hard.

**Results:**

Twenty-seven studies were included in the meta-analysis. No difference was found between coaches and athletes for overall RPE (SMD = 0.19, *P* = 0.10) and overall sRPE (SMD = 0.05, *P* = 0.75). There was a difference for easy RPE (SMD = − 0.44, small effect size, *P* = 0.04) and easy sRPE (SMD = − 0.54, moderate effect size, *P* = 0.04). No differences were found for moderate RPE (SMD = 0.05, *P* = 0.74) and hard RPE (SMD = 0.41, *P* = 0.18). No difference was found for moderate (SMD = -0.15, *P* = 0.56) and hard (SMD = 0.20, *P* = 0.43) sRPE.

**Conclusion:**

There is an agreement between coaches and athletes about overall RPE and sRPE, and RPE and sRPE into two effort categories (moderate and hard). However, there were disagreements in RPE and sRPE for easy effort category. Thus, despite a small disagreement, the use of these tools seems to be adequate for training monitoring.

**Supplementary Information:**

The online version contains supplementary material available at 10.1186/s40798-022-00420-3.

## Key Points

- The session rating of perceived exertion (sRPE) may be used to indicate the internal training load in several sports.
- The ratings of perceived exertion (RPE) and sRPE in easy sessions prescribed/intended/observed by coaches may underestimate athletes' RPE/sRPE.
- The RPE and sRPE may guide coaches' and sport scientists' decision-making in training programming in several sports.

## Introduction

Several studies have shown the importance of training load monitoring in various sports modalities [1–3]. High training loads without adequate recovery may trigger unwanted adaptations and negative results, whereas loads with insufficient duration and intensities may not generate necessary adaptations to improve physical performance [4, 5]. In this sense, precise training load control and manipulation are required [6]. Traditionally, training load has been measured by power, velocity, acceleration, movement repetition count, and global positioning system (GPS) parameters [6]. This way of measuring the athletes' training load has been called external load. In turn, the emerging literature has measured the physiological stress imposed on the athlete during training or competitions, defined as internal load. Heart rate, blood lactate, oxygen consumption, rating of perceived exertion (RPE), and session RPE (sRPE) have been widely used to verify the internal load in athletes [7].

The use of technological tools to control training load (heart rate monitors, GPS, smartphone apps, etc.) is a reality of contemporary training monitoring. However, the large amount of information can become a real problem for coaches' analysis. Additionally, using these equipments involves a cost that varies widely from hundreds to thousands of dollars [8]. These values increase when we need to monitor many athletes simultaneously. Foster et al. [9] recommend keeping it simple, which may be the most crucial element of training monitoring. Thus, the use of RPE and sRPE is considered an easy-to-use, non-invasive, accessible, valid, and reliable method for coaches to assess the training load applied to athletes daily, improving the control of training variables [2, 10–13].

The sRPE method uses an objective measure of training load (time) interacting with a subjective one (RPE), thus giving a training load index in arbitrary units (a.u.) [2, 14] extensively accepted as a marker of the internal training load [13]. In addition, the sRPE has been used to assess the agreement between coach and athlete for load planned and perceived [5]. Previous studies [15, 16] have shown good agreement between coaches and athletes. For instance, Redkva et al. [16] found no differences when comparing the sRPE prescribed by the coaches and perceived by the athletes in physical, technical, and tactical training sessions during the three weeks of pre-season in soccer players. However, some studies [5, 17–19] have reported a difference between the training load planned by coaches and the load perceived by athletes. In general, these disagreements between coaches and athletes were identified in the prescribed training as easy or hard [5, 17–19]. Besides, Rabelo et al. [20] demonstrated that in all three effort categories (easy, moderate, and hard), the athletes perceived a lower training load than intended by the coach. Foster et al. [17], based on empirical observations, suggest that this lack of correspondence between the program planned by the coach and that carried out by the athletes is a potential cause of the high incidence of negative results in sports training. In this scenario, incorrect interpretation of sRPE data can lead to errors in the control and subsequent planning of training. The training program is prescribed to balance overload and recovery [2], determining the positive or negative adaptation of the training stimulus. It is known that too low a training load can result in detraining status [21]. On the other hand, too high a training load and poor recovery can result in overtraining syndrome [4, 22] and developing overuse injuries [23]. Therefore, the balance between training load and recovery represents a significant challenge for coaches and athletes.

We acknowledge that recent studies investigated the internal load in several sports (for reviews, see [3, 6]). However, no systematic review and meta-analysis were performed to elucidate and summarize the differences between the internal load perceived by the athletes and that planned/intended/observed by the coaches. Giving daily control with feedback to coaches is the key to improving physical performance and decreasing the risk of injuries and harmful effects of training. An example is manipulating the future training load to re-align with the planned load [20]. Coaches should be aware that athletes could interpret the same training differently. A simple and subjective method to quantify the internal load of the designed and executed training programs could serve as a tool to optimize the training process [9, 13]. Therefore, this systematic review and meta-analysis of the literature aimed to investigate whether there are differences between the training load perceived by athletes and that prescribed/intended/observed by coaches. We hypothesized that significant differences would be found between that expected by coaches and that perceived by the athletes [5, 17–19].

## Methods

### Protocol and Registration

This systematic review and meta-analysis was written according to the Preferred Reporting Items for Systematic Reviews and Meta-Analysis (PRISMA) [24]. The protocol was registered in the Open Science Framework in September 2020, with storage in Australia-Sydney (Available at: osf.io/wna4x).

### Search Strategy

A systematic literature search was conducted in September 2020 and updated in November 2021. The following databases were used: PubMed, Web of Science, Embase, and SPORTDiscus. The following descriptors were used: “SRPE,” OR “Session rating of perceived exertion,” OR “Session RPE,” OR “Session-RPE,” OR “Training dose,” OR “Ratings of perceived exertion,” OR “Training load,” OR “Training loads,” OR “Internal load,” OR “Internal training load,” OR “External load,” OR “External training load,” AND “Coaches,” OR “Coach,” OR “Mismatch between coaches-players perceptions,” OR “Coaches-players perceptions,” OR “Discrepancy between coach-athlete perceptions,” OR “Comparison of athlete-coach perceptions,” OR “Impaired player-coach perceptions,” OR “Relationship between coach-athlete perceptions.” In addition, the reference lists were explored to find additional relevant studies. No filters were applied in the search as a limitation of time and language.

### Eligibility Criteria

Eligibility criteria for study inclusion consisted of one of the following: (a) Population: athletes and coaches of any sport (individual or team), sex, age, or experience level; (b) Comparison: between the training/competition (all season periods) load prescribed/intended/observed by the coaches and that performed/reported/perceived by the athletes; (c) Outcome: RPE or sRPE for any scale considering overall training/competition sessions (physical, strength, tactical, technical, games) and/or classified into three effort categories: easy, moderate and hard. These three effort categories were adopted because they are the three intensity zones typically used in the literature related to the comparison of RPE/sRPE between coaches and athletes [5, 17–20, 25–34]; (d) Study design: cross-sectional studies published in peer-reviewed journals. Conference abstracts, dissertations, theses, book chapters, and articles published in non-peer-reviewed journals were not included.

### Study Selection

Study eligibility assessments were performed independently by two reviewers (AI and PB). First, studies were downloaded from EndNote (version X9.0, Clarivate Analytics, Philadelphia, PA, USA), and duplicates were removed before being selected by title and abstract. Then, the full texts of the remaining studies were retrieved and evaluated for eligibility. Any disagreements regarding the inclusion of a particular study were resolved through a consensus meeting. When there was no consensus, the third researcher (ES) decided whether to include or exclude the study.

### Data Extraction

The following data were extracted from the articles: participant characteristics, sample size, training level, type of sports, the scale used, intensity zones, number of sessions/training duration, number of coaches, coaches' experience time, number of athletes, and results. Importantly, data extraction from the selected studies was processed independently by two researchers (AI and PB). Differences were resolved through a consensus meeting or a third reviewer (ES) decision. When the data to be extracted were not found, the principal authors were contacted. When no response was obtained from the principal authors, the data imputation technique using the Kinovea 0.8.15 software was used to extract data reported in figures.

### Assessment of Methodological Quality

To assess the methodological quality of the included studies, we used the Quality Assessment Tools for Observational Cohort and Cross-Sectional Studies (Available in: https://www.nhlbi.nih.gov/health-topics/study-quality-assessment-tools). Reviewers answered each question as “Yes,” “No,” “Cannot determine,” “Not applicable,” or “Not reported,” based on the critical review of each study. Questions answered with “Yes” received a score of 1, while questions answered with “No,” “Cannot determine,” or “Not reported” received a score of 0. The total score for each study was used to rank the risk of bias as low (6–8), moderate (3–5), or high (0–2). The methodological quality assessment was performed independently by two experienced evaluators (AI and PB). Any disagreements were resolved through a consensus meeting or a third reviewer (ES) decision.

### Certainty of Evidence

Two evaluators (AI and PB) independently assessed the certainty of evidence using the Grading of Recommendations Assessment, Development and Evaluation (GRADE) approach [35] through the GRADE PRO website (https://gradepro.org). GRADE specifies four categories: high, moderate, low, and very low, applied to a body of evidence [35]. The observational studies included in this review started with low certainty of evidence. Five aspects can decrease the certainty of evidence (a) risk of bias (decreased if more than 25% of participants were from studies with a moderate or high risk of bias); (b) inconsistency of results (decreased if heterogeneity *I*^2^ > 50%); (c) indirect evidence (decreased if the outcomes evaluated are not those of primary interest); (d) imprecision (decreased if less than 140 participants were included in the comparison) and (e) other (e.g., publication bias). Three aspects can increase the certainty of evidence (a) effect size (increased if large effect size), (b) dose–response gradient (increased if study effect size increases due to an increase in an independent variable), and (c) confounding factors (increased if the main potential confounding variables were measured and adjusted statistically). For each aspect that met the criterion, the certainty was increased by one level. If the criterion was not satisfied, the certainty was decreased by one level [35]. Any disagreements were resolved through a consensus meeting or a third reviewer (ES) decision.

### Statistical Analysis

A meta-analysis was performed through the Review Manager software (RevMan Version 5.4; the Nordic Cochrane Centre, Copenhagen: Cochrane Collaboration). Each  standardized mean difference (SMD) was weighted according to the inverse variance method. The SMD values in each trial were pooled with a random (if heterogeneity was significant) or fixed-effects model (if heterogeneity was by chance). SMD values of 0.2, 0.5, and 0.8 represent small, moderate and large effect sizes, respectively [36]. Heterogeneity between studies was assessed using I^2^ statistics. I^2^ values between 0–50% represent low heterogeneity, between 50 and 74% moderate heterogeneity and ≥ 75% high heterogeneity [37]. Funnel plots and Egger’s regression analysis were also performed using StatsDirect software (Version 3). They were used to assess publication bias. Statistical significance was set at 5% (*P* ≤ 0.05).

## Results

### Selection of Studies

**Fig. 1:**
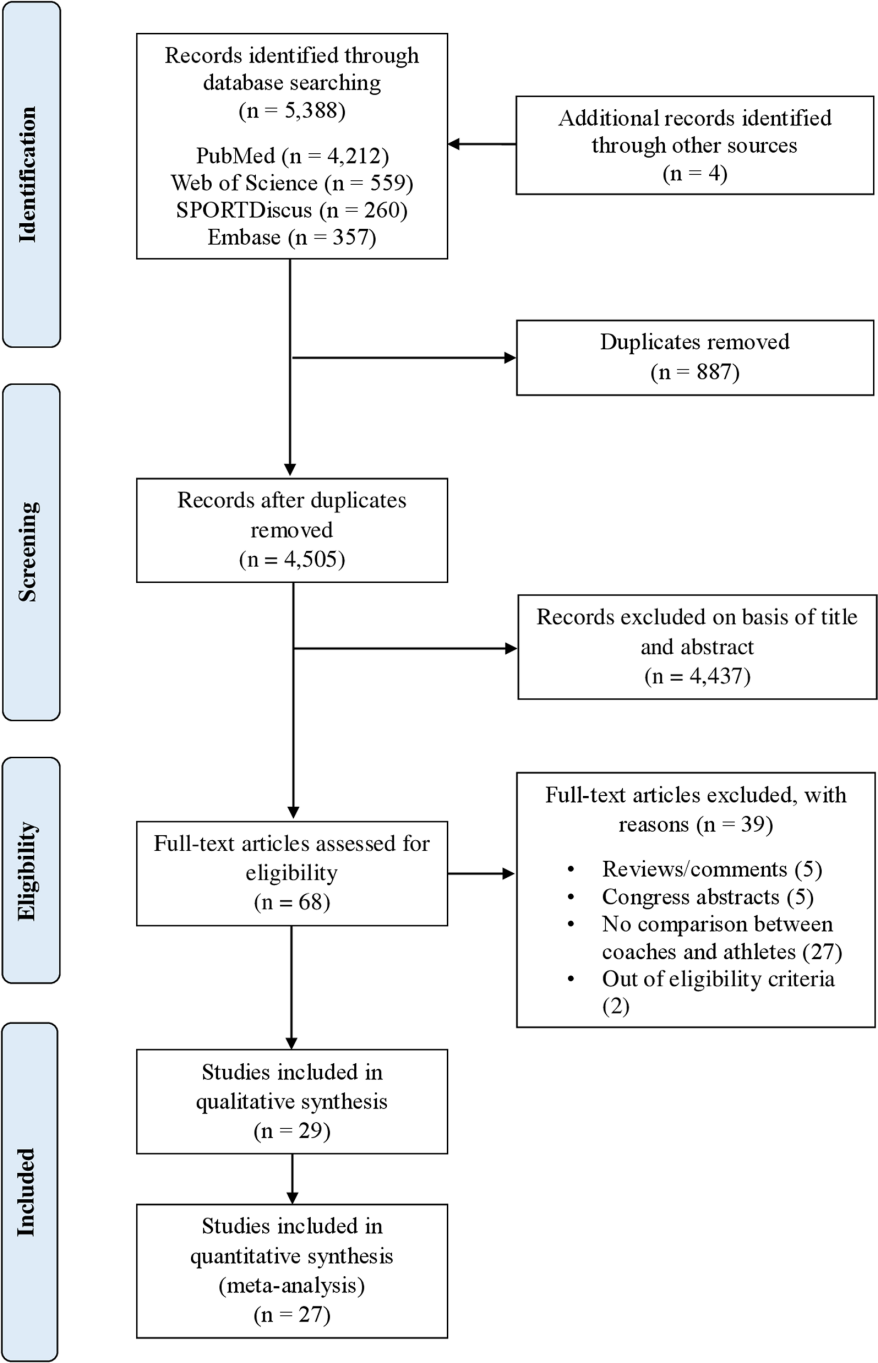
Literature search flowchart

The results identified a total of 5,388 articles. Four additional records were identified through direct citation search and manual verification of article reference lists. After removing duplicate reports (*n* = 887), a total of 4505 articles remained. A summary of the search results and reasons for exclusion are shown in Fig. 1. Twenty-nine studies were included in this systematic review and 27 in meta-analysis since two studies failed to report the standard deviation of the coach rating of exertion. Data are summarized in Table 1.

**Table 1:** Summary of the included studies

Study	Participant characteristics				Sample size	Type of sport	Training content and metrics	Scale and intensity zones	Number of coaches and/or yrs experience	Results
	Age (years)	Stature (cm)	Body mass (kg)	Status/level	M/F/T					
Andrade et al. [44]	23.2 ± 2.2	180 ± 10	79.0 ± 6.0	Elite (Brazilian Open Circuit, World Championships and Olympic Games)	0/3/3	Beach Volleyball	3 weeks; Overall RPE and sRPE	CR10	2 coaches; 1 tactical-technical and 1 physical fitness (> 15 yrs of professional coaching experience)	**RPE** 1st week: Coaches > Athletes (Likely difference) 2nd week: Coaches = Athletes 3rd week: Coaches = Athletes Physical fitness training sessions: Coaches > Athletes (Likely difference) Strength training sessions: Coaches > Athletes (Likely difference) Tactical-technical training sessions: Coaches = Athletes **sRPE** 1st week: Coaches > Athletes 2nd week: Coaches = Athletes 3rd week: Coaches = Athletes Physical fitness training sessions: Coaches < Athletes (Probable difference) Strength training sessions: Coaches > Athletes (Probable difference) Tactical-technical training sessions: Coaches = Athletes
Andrade Nogueira et al. [25]	24 ± 2.8	189.3 ± 9.7	84.6 ± 11.1	Professional (Brazilian first division league)	15/0/15	Volleyball	34 training sessions; 2 (5.9%) easy, 23 (67.6%) moderate and 9 (26.5%) hard sessions; RPE for easy, moderate and hard zones	CR10 RPE Easy < 3 Mod. 3–5 Hard > 5	1 coach	**RPE** Easy zone: Coaches < Athletes (*P* < 0.01) Moderate zone: Coaches < Athletes (*P* < 0.01) Hard zone: Coaches > Athletes (*P* < 0.01)
Barnes et al. [19]	M: 20.2 ± 1.4; F: 19.7 ± 1.6	M: 176.6 ± 7.8; F: 168.4 ± 6.5	M: 67.9 ± 7.1; F: 53.9 ± 6.0	Highly trained	13/12/25	Cross-country runners	110 training days (3024 sessions; 1875 (62%) easy, 544 (18%) moderate and 605 (20%) hard sessions); RPE and sRPE for overall, easy, moderate and hard zones	CR10 RPE Easy < 3 Mod. 3–5 Hard > 5	2 coaches	**RPE** Easy zone: Coach < M athlete (*P* < 0.0001), and F athlete (*P* < 0.001) Moderate zone: Coach < M athlete (*P* = 0.001); Coach = F athlete (*P* = 0.38) Hard zone: Coach = M athlete (*P* = 0.91); Coach > F athlete (*P* = 0.006) **sRPE** Easy zone: Coach < M athlete (*P* < 0.0001), and F athlete (*P* < 0.001) Moderate zone: Coach < M athlete (*P* = 0.002); Coach = F athlete (*P* = 0.61) Hard zone: Coach = M athlete (*P* = 0.68); Coach > F athlete (*P* = 0.008)
Barroso et al. [26]	11–12 yrs: 11.2 ± 0.4; 13–14 yrs: 13.4 ± 0.5; 15–16 yrs: 15.4 ± 0.6	11–12 yrs: 146.7 ± 4,5; 13–14 yrs: 158.4 ± 6.9; 15–16 yrs: 169.6 ± 6.1	11–12 yrs: 38.8 ± 2.4; 13–14 yrs: 49.6 ± 5.4; 15–16 yrs: 60.1 ± 5.8	Local, State and National level	NR/NR/160	Swimming	9 training sessions; NR n easy, NR n moderate and NR n hard sessions; RPE for easy, moderate and hard zones	CR10 RPE Easy < 3 Mod. 3–5 Hard > 5	9 coaches; 11–12 yrs: 13.2 ± 1.9 yrs of competitive coaching experience; 13–14 yrs: 12.1 ± 1.7 yrs of competitive coaching experience; 15–16 yrs: 14.5 ± 2.7 yrs of competitive coaching experience	**RPE (range 11–12 years old)** Easy and moderate for Athletes > Coaches (*P *< 0.05) Hard for Athletes < Coaches (*P* < 0.05) **RPE (range 13–14 yrs old)** Easy and moderate for Athletes > Coaches (*P* < 0.05) Hard for Athletes < Coaches (*P* < 0.05) **RPE (range 15–16 yrs old)** Easy and moderate for Athletes = Coaches (*P* > 0.05) Hard for Athletes < Coaches (*P* < 0.05) **Correlations** Range 11–12 yrs old: *r* = 0.31 (*P* < 0.001) Range 13–14 yrs old: *r* = 0.51 (*P* < 0.001) Range 15–16 yrs old: *r* = 0.74 (*P* < 0.001)
Barroso et al. [38]	21.1 ± 1.1	178 ± 6	74.1 ± 8.3	Moderately trained	13/0/13	Swimming	4 training sessions; Overall RPE	CR10	1 coach (7 yrs coaching experience)	**RPE** Overall: Coach > Athletes (10 × 200 m (*P* = 0.005) and 5 × 400 m (*P* = 0.033)
Brink et al. [5]	U17; U19	U17: 174.5 ± 7.9 U19: 178.3 ± 7.1	U17: 61.7 ± 6.5 U19: 71.1 ± 8.6	Professional	33/0/33	Soccer	2446 training sessions; NR n easy, NR n moderate and NR n hard sessions; RPE and sRPE for overall, easy, moderate and hard zones	6 to 20 Borg scale RPE Easy < 13 Mod.13–14 Hard > 14	2 coaches; (numerous yrs of experience at the highest level -UEFA A and PRO license)	**RPE** Overall: Coaches < Athletes (*P* < 0.0001) Easy zone: Coaches < Athletes (*P* < 0.0001) Moderate zone: Coaches < Athletes (*P* < 0.0001) Hard zone: Coaches > Athletes (*P* < 0.0001) **sRPE** Overall: Coaches < Athletes (*P* < 0.0001) Easy zone: Coaches < Athletes (*P* < 0.0001) Moderate zone: Coaches < Athletes (*P* < 0.0001) Hard zone: Coaches > Athletes (*P* < 0.0001) **Duration** Overall: Coaches = Athletes (*P* > 0.05) Easy zone: Coaches = Athletes (*P* > 0.05) Moderate zone: Coaches = Athletes (*P* > 0.05) Hard zone: Coaches > Athletes (*P* < 0.0001) **Correlations** RPE Coaches and Athletes: *r* = 0.24 (*P* < 0.0001) sRPE Coaches and Athletes: *r* = 0.41 (*P* < 0.0001) Duration Coaches and Athletes: *r* = 0.49 (*P* < 0.0001)
Brink et al. [39]	U15: 14.3 ± 0.3 U17: 16.3 ± 0.2	U15: 168.1 ± 11.1 U17: 179.9 ± 4.9	U15: 56.3 ± 12.9 U17: 67.8 ± 5.2	Highest level of competition in the Netherlands	31/0/31	Soccer	977 training sessions (8 weeks); U15 = 445 sessions; U17 = 532 sessions; Overall RPE, RIE and ROE	6 to 20 Borg scale	2 coaches; 1 U15 coach = 18 yrs of professional coaching experience; 1 U17 coach = 23 yrs of professional coaching experience	**RPE** Coaches RIE and ROE < Athlete's RPE (*P* < 0.01) **Correlations** Coaches RIE and Athlete's RPE: *r* = 0.58 (*P* < 0.01) Coaches ROE and Athlete's RPE: *r* = 0.64 (*P* < 0.01)
Cruz et al. [27]	14.0 ± 1.32	162.7 ± 9.3	52.4 ± 11.5	State level	15/13/28	Track and field	4 weeks (20 training sessions); NR n easy, NR n moderate and NR n hard sessions; RPE for overall, easy, moderate and hard zones	CR10 RPE Easy < 3 Mod. 3–5 Hard > 5	1 coach (6 yrs of experience)	**RPE** Overall: Coach > Athletes (*P* < 0.001) Easy zone: Coach > Athletes (*P* < 0.001) Moderate zone: Coach > Athletes (*P* < 0.001) Hard zone: Coach < Athletes (*P* < 0.001)
Doeven et al. [40]	26.7 ± 3.8	197.2 ± 9.1	100.3 ± 15.2	Elite	14/0/14	Basketball	15 matches within 6 weeks (2.5 matches/week); Overall RPE, ROE and sRPE	6 to 20 Borg scale	3 coaches: 1 head coach (> 10 yrs of elite coaching experience), 1 assistant coach and 1 strength and conditioning coach	**RPE** Coaches ROE > Athlete's RPE (*P* = 0.029) **Correlations** Coaches ROE and Athlete's RPE: *r* = 0.25 (*P* < 0.01)
Figueiredo et al. [15]	18.7 ± 0.7	175.3 ± 5.5	68.7 ± 6.5	Elite U19	16/0/16	Soccer	3 weeks (15 training sessions, 5 training sessions (1 training session per day) per week); Overall RPE and sRPE	CR10	2 coaches: 1 technical coach and 1 fitness coach	**RPE** Athletes = Coaches (*P* = 0.62) **sRPE** Athletes = Coaches (*P* = 0.86) **Correlations (sRPE)** Coaches and Athletes: *r* = 0.84 (*P* < 0.001)
Foster et al. [17]	NR	NR	NR	Competitive	6/9/15	Middle and long distance runners	5 weeks training sessions; NR n easy, NR n moderate and NR n hard sessions; RPE and sRPE for easy, moderate and hard zones	CR10 RPE Easy < 3 Mod. 3–5 Hard > 5	3 coaches (each athlete had only one coach)	**RPE** Easy zone: Coaches < Athletes (*P* < 0.05) Moderate zone: Coaches = Athletes (*P* > 0.05) Hard zone: Coaches > Athletes (*P* < 0.05) **sRPE** Easy zone: Coaches < Athletes (*P* < 0.05) Moderate zone: Coaches = Athletes (*P* > 0.05) Hard zone: Coaches > Athletes (*P* < 0.05) **Duration** Easy zone: Coaches = Athletes (*P* > 0.05) Moderate zone: Coaches = Athletes (*P* > 0.05) Hard zone: Coaches = Athletes (*P* > 0.05) **Correlations** RPE Athletes and Coaches: *r* = 0.75 (*P* ≤ 0.05) sRPE Athletes and Coaches: *r* = 0.74 (*P* ≤ 0.05) Duration Athletes and Coaches: *r* = 0.65 (*P* ≤ 0.05)
Ieno et al. [28]	23.75 ± 4.86	167 ± 6.68	62.25 ± 6.16	Elite (Olympic team)	2/2/4	Open-water swimmers	5 weeks (160 training sessions); (37.8% (60) easy, 44.1% (71) moderate and 18.1% (29) hard sessions); RPE, for easy, moderate and hard zones	CR10 RPE Easy ≤ 3 Mod. 4–6 Hard ≥ 7	1 coach	**RPE** Easy zone: Coach = Athletes (*P* = 0.663) Moderate zone: Coach = Athletes (*P* = 0.110) Hard zone: Coach = Athletes (*P* = 0.149)
Imamura et al. [41]	Black belt: 21.5 ± 0.5 White belt: 19.9 ± 0.8	Black belt: 171.8 ± 7.4 White belt: 169.4 ± 4.6	Black belt: 66.8 ± 8.9 White belt: 59.9 ± 7.3	Fukuoka University Karate Club (Black belt: Japan National Karate Team and top competitors in Japan)	14/0/14	Karate	1 session (1000 punches and 1000 kicks); Overall RPE	6 to 20 Borg scale	9 coaches	**RPE** 1000 punches (black belt and white belt) and 1000 kicks (black belt): Coach > Athletes (*P* < 0.05) 1000 kicks (white belt): Coach = Athletes (*P* > 0.05)
Inoue et al. [29]	33.1 ± 9.9	177.3 ± 6.7	71.1 ± 6.9	Trained	14/0/14	Road Cycling and Mountain Biking	2 weeks during the competitive period (146 training sessions); 11.6% (17) easy, 59.6% (87) moderate and 28.8% (42) hard sessions; RPE and sRPE for overall, easy, moderate and hard zones	CR10 RPE Easy < 3 Mod. 3–5 Hard > 5	5 coaches (7.2 ± 4.1 yrs of coaching experience)	**RPE** Overall: Coaches = Athletes (*P* = 0.586) Easy zone: Coaches < Athletes (*P* = 0.013) Moderate zone: Coaches = Athletes (*P* = 0.651) Hard zone: Coaches = Athletes (*P* = 0.051) **sRPE** Overall: Coaches = Athletes (*P* = 0.738) Easy zone: Coaches = Athletes (*P* = 0.073) Moderate zone: Coaches = Athletes (*P* = 0.664) Hard zone: Coaches = Athletes (*P* = 0.388) **Duration** Overall: Coaches = Athletes (*P* = 0.717) Easy zone: Coaches = Athletes (*P* = 0.393) Moderate zone: Coaches = Athletes (*P* = 0.980) Hard zone: Coaches = Athletes (*P* = 0.853) **Correlations** RPE Coaches and Athletes: *r* = 0.73 (*P* < 0.0001) sRPE Coaches and Athletes: *r* = 0.87 (*P* < 0.0001) Duration Coaches and Athletes: *r* = 0.95 (*P* < 0.0001)
Kraft et al. [30]	18–23	NR	NR	4 Division intercollegiate athletic teams	NR/NR/56	Volleyball, Basketball, and Soccer	433 training sessions (~ 45% (195) easy, ~ 48% (208) moderate and ~ 7% (30) hard sessions); RPE, RIE and ROE for overall, easy, moderate and hard zones	Generic 0 to 10 point scale using the Omni verbal cues for adults RPE Easy ≤ 4 Mod. 5–7 Hard ≥ 8	4 head coaches	**RPE** Overall: Athlete's RPE < Coaches RIE, and ROE (*P* ≤ 0.05) Easy zone: Athlete's RPE < Coaches ROE (*P* ≤ 0.05) Moderate zone: Athlete's RPE < Coaches ROE (*P* ≤ 0.05) Hard zone: Athlete's RPE > Coaches ROE (*P* ≤ 0.05) **Correlations** Athlete's RPE and Coaches ROE: *r* = 0.83 (*P* ≤ 0.05) Athlete's RPE and Coaches RIE: *r* = 0.65 (*P* ≤ 0.05)
Lupo et al. [45]	16.7 ± 0.5	178 ± 9	72 ± 9	Elite	0/15/15	Basketball	268 training sessions; Overall sRPE	CR10	4 coaches (1head and 2 assistant coaches, 1 physical trainer)	**sRPE** Strength training sessions: Coaches = Athletes (*P* > 0.05) Conditioning training sessions: Coaches > Athletes (*P* = 0.01) Technique training sessions: Coaches < Athletes (*P* < 0.001)
Medina et al. [42]	27 ± 5.1	175.9 ± 5.9	73.9 ± 6.1	Professional	12/0/12	Futsal	40 weeks (225 training sessions); Overall RPE	6 to 20 Borg scale	2 coaches	**RPE** Coaches = Athletes **Correlations** Coaches and Athletes: *r* = 0.74 (*P* < 0.05)
Murphy et al. [49]	15.0 ± 1.2	167.0 ± 10.8	60.0 ± 14.2	Elite junior	8/6/14	Tennis	21 ± 3 training sessions (16 weeks, 285 drills); RPE, drill physical RPE, drill mental RPE	CR10	6 coaches (10 ± 3 yrs elite-level coaching experience)	**RPE** Coaches < Athletes (*P* < 0.01) **Correlations** Coaches and Athletes: *r* = 0.59 (*P* < 0.05)
Nogueira et al. [31]	15.2 ± 0.57	170.1 ± 6.3	59.7 ± 5.7	State and National	10/7/17	Swimming	18 training sessions; 11.1% (2) easy, 83.3% (15) moderate and 5.6% (1) hard sessions; RPE for overall, easy, moderate and hard zones	CR10 RPE Easy < 3 Mod. 3–5 Hard > 5	1 coach	**RPE** Overall: Coaches = Athletes (*P* > 0.05) Easy zone: Coaches = Athletes (*P* > 0.05) Moderate zone: Coaches = Athletes (*P* > 0.05) Hard zone: Coaches = Athletes (*P* > 0.05)
Oytun et al. [46]	20.7 ± 5.4	164.0 ± 6.7	62.8 ± 10.0	First division women’s handball league in North Cyprus	0/56/56	Handball	57 training sessions, Overall sRPE	CR10	4 coaches	**sRPE** Coaches > Athletes (*P* = < 0.02) **Correlations** Coaches and Athletes: *r* = 0.93 (*P* < 0.01)
Rabelo et al. [20]	24.6 ± 3.8	176 ± 10	77.0 ± 9.6	Professional	18/0/18	Futsal	45 weeks (157 training sessions); NR n easy, NR n moderate and NR n hard sessions; RPE for easy, moderate, hard zones and season periods	CR10 RPE 2-step cluster to classify easy, moderate and hard zones	1 coach (> 3 yrs experience)	**RPE** Coaches > Athletes (all season periods (Pre-season, first competitive period, intercompetition period and second competitive period) and intensity zones (easy, moderate and hard))
Redkva et al. [16]	24.1 ± 3.4	178.6 ± 6.1	78.3 ± 9.2	Professional	24/0/24	Soccer	22 training sessions (1465 min, 32% (470 min) = physical, 20% (290 min) = technical and 48% (705 min) = tactical training); First 3 weeks of preseason; Overall RPE and sRPE	CR10	2 coaches: 1 technical coach (8 yrs coaching experience), 1 fitness coach (20 yrs experience)	**sRPE** Overall: Coaches = Athletes (*P* = 0.70) Tactical training sessions: Coaches = Athletes (*P* > 0.05) Physical training sessions: Coaches = Athletes (*P* > 0.05) Technical training sessions: Coaches = Athletes (*P* > 0.05) **Correlations** Coaches and Athletes: *r* = 0.60 (*P* < 0.003)
Rodrigues-Marroyo et al. [47]	21 ± 3	170.5 ± 6.6	63.7 ± 4.9	University team (Spanish volleyball third division)	0/12/12	Volleyball	15 weeks of training; Overall RPE and sRPE	CR10	4 coaches (2 experts (> 10 yrs experience), 2 beginners (≤ 1 yr experience)	**RPE** Overall: Coaches (experts and beginners) = Athletes (*P* > 0.05) **sRPE** Overall: Coaches (experts and beginners) = Athletes (*P* > 0.05) **Correlations** RPE Expert coaches and Athletes: *r* = 0.70 (*P* < 0.01) RPE Beginner coaches and Athletes: *r* = 0.72 (*P* < 0.01) sRPE Expert coaches and Athletes: *r* = 0.75 (*P* < 0.01) sRPE Beginner coaches and Athletes: *r* = 0.76 (*P* < 0.01)
Scantlebury et al. [32]	Hockey: 17.4 ± 0.8; Netball: 17.6 ± 0.6; Rugby union: 17.2 ± 0.4 Soccer: 17.2 ± 0.8	Hockey: 164.7 ± 6.4; Netball: 167.8 ± 4.2; Rugby union: 79.9 ± 5.4; Soccer: 174.0 ± 0.05	Hockey: 60.0 ± 6.3; Netball: 58.0 ± 7.2; Rugby union: 83.6 ± 11.5 Soccer: 73.6 ± 7.1	Independent school	20/17/37	Hockey, Netball, Rugby union, and Soccer	219 training sessions; 28 easy, 125 moderate and 66 hard sessions; RPE, RIE and ROE for overall, easy, moderate and hard zones	CR10 RPE Easy < 3 Mod. 3–4 Hard ≥ 5	4 coaches (1 per sport). All coaches had > 5 yrs coaching experience and had worked with participants for > 1 yr	**RPE** Overall: Athlete's RPE = Coaches RIE, and ROE Easy zone: moderate ≠ between Athlete's RPE and Coaches RIE (ES: 1.17; 95% CI 0.7 to 1.65) and Coaches ROE (ES: 0.83; 95% CI 0.4 to 1.28) Moderate zone: small ≠ between Athlete's RPE and Coaches RIE (ES: -0.36; 95% CI -0,56 to -0.11) and Coaches ROE (ES: -0.29; 95% CI -0.46 to -0.11) Hard zone: small ≠ between Athlete's RPE and Coaches RIE (ES: -0.46; 95% CI -0,72 to -0.20); trivial ≠ between Athlete's RPE and Coaches ROE (ES: -0.05; 95% CI -0.24 to 0.36) **Correlations** Athlete's RPE and Coaches RIE: *r* = 0.39 (95% CI 0.27 to 0.49) Athlete's RPE and Coaches ROE: *r* = 0.63 (95% CI 0.54 to 0.70)
Sinnott-O’Connor et al. [33]	19 ± 4	NR	48.5 ± 7.6	World class (Irish Paralympic swimming team)	1/3/4	Paralympic Swimmers	16 training sessions; NR n easy, NR n moderate and NR n hard sessions; sRPE for easy, moderate and hard zones	CR10 RPE Easy < 3 Mod. 3–5 Hard > 5	1 coach	**sRPE** Easy zone: Coach < Athletes (*P* ≤ 0.05) Moderate zone: Coach < Athletes (*P* ≤ 0.01) Hard zone: Coach < Athletes (*P* ≤ 0.05)
Vaquera et al. [43]	16 ± 0.4	183.9 ± 5.8	NR	Junior Spanish national League	12/0/12	Basketball	6 weeks (15 training sessions); 7 games of one-a-side (1v1), 6 games of 2-a-side (2v2), 8 games of 5-a-side (5v5), and 5 games of superiority (3v2) situations; Overall RPE	CR10	1 coach (18 yrs experience training U16 and U18 players)	**RPE** Coach < Athletes (in all games, all *P* < 0.0001 apart from 5v5 *P* = 0.0019)
Viveiros et al. [48]	NR	NR	NR	Brazilian national team	NR/NR/40	Judo	4 training sessions; Overall RPE	CR10	4 separate coaches for the sessions	**RPE** Overall: Coach < Athletes (*P* < 0.05)
Voet et al. [34]	M: 21 ± 3; F: 19 ± 0	NR	M: 69.1 ± 4.4; F: 60.8 ± 1.1	Semi-professional 2 females and 1 male cyclist (World Tour team)	9/2/11	Road Cycling	747 training sessions; NR n easy, NR n moderate and NR n hard sessions; RPE and sRPE for overall, easy, moderate and hard zones	6 to 20 Borg scale RPE Easy < 11 Mod. 11–14 Hard > 14	1 coach (5 yrs experience in coaching cyclists at UCI Continental level, or higher)	**RPE** Overall: Coach = Athletes (*P* > 0.05) Easy zone: Coach > Athletes (*P* = 0.004) Moderate zone: Coach = Athletes (*P* > 0.05) Hard zone: Coach > Athletes (*P* < 0.001) **sRPE** Overall: Coach = Athletes (*P* > 0.05) Easy zone: Coach = Athletes (*P* > 0.05) Moderate zone: Coach = Athletes (*P* > 0.05) Hard zone: Coach = Athletes (*P* > 0.05) **Duration** Overall: Coach = Athletes (*P* > 0.05) **Correlations** RPE Coach and Athletes: *r* = 0.73 (*P* < 0.05) sRPE Coach and Athletes: *r* = 0.87 (*P* < 0.05)
Wallace et al. [18]	22.3 ± 3.1	175.0 ± 9.0	71.8 ± 11.6	Well-trained (National level)	6/6/12	Swimming	3-month period (20 individual training sessions); NR n easy, NR n moderate and NR n hard sessions; RPE and sRPE for easy, moderate and hard zones	CR10 RPE Easy < 3 Mod. 3–5 Hard > 5	2 coaches (qualified swimming instructors)	**RPE** Easy zone: Coaches < Athletes (*P* < 0.05) Moderate zone: Coaches = Athletes (*P* > 0.05) Hard zone: Coaches > Athletes (*P* < 0.05) **sRPE** Easy zone: Coaches = Athletes (*P* > 0.05) Moderate zone: Coaches = Athletes (*P* > 0.05) Hard zone: Coaches = Athletes (*P* > 0.05) **Correlations** RPE Coaches and Athletes: *r* = 0.84 (*P* < 0.01) sRPE Coaches and Athletes: *r* = 0.85 (*P* < 0.01) Duration Coaches and Athletes: *r* = 0.86 (*P* < 0.01)

NR = not reported; M = males; F = females; yrs = years; T = total; >  = greater-than; <  = less-than; ≠  = difference; n = number of easy, moderate and hard sessions; Mod. = moderate; ES = effect size; 95% CI = 95% confidence interval; U = under; UCI = *Union Cycliste Internationale*; 6–20 Borg scale = 6 to 20 Borg ratings of perceived exertion scale; CR10 = category ratio 10 scale; RPE = ratings of perceived exertion; sRPE = session ratings of perceived exertion; RIE = ratings of intended exertion by coaches; ROE = ratings of observed exertion by coaches

### Study Characteristics

This review contains 725 participants (306 men, 163 women, and 256 unspecified). Of the 29 studies included in the review, 12 studies included only male participants [5, 15, 16, 20, 29, 31, 38–43], four studies included only female participants [44–47], three studies did not specify the sex of the participants [26, 30, 48], and 10 studies included a combination of male and female participants [17–19, 27, 28, 31–34, 49].

All 29 studies recruited only athletes. The evaluated sports were swimming (*n* = 4), soccer (*n* = 4), tennis (*n* = 1), middle and long distance running (*n* = 1), cross-country running (*n* = 1), beach volleyball (*n* = 1), volleyball (*n* = 2), track and field (*n* = 1), basketball (*n* = 3), open-water swimmers (*n* = 1), karate (*n* = 1), road cycling and mountain biking (*n* = 1), futsal (*n* = 2), handball (*n* = 1), paralympic swimmers (*n* = 1), judo (*n* = 1), road cycling (*n* = 1), and combination of different sports (*n* = 2) including volleyball, basketball, soccer, hockey, netball and rugby union.

The studies were published between the years 1997 to 2021. The number of training sessions ranged from one to 3024. The number of coaches ranged from 1 to 9. Of the 29 studies included in the review, eight studies included two coaches [5, 15, 16, 18, 19, 39, 42, 44], two studies had three coaches [17, 40], six studies included four coaches [30, 32, 45–48], one study included five coaches [29], one study included six coaches [49], and two studies included nine coaches [26, 41]. The coaches' experience ranged from ≤ 1 year to > 23 years. Fourteen studies did not report the length of experience of coaches [15, 17–19, 25, 28, 30, 31, 33, 41, 42, 45, 46, 48].

### Differences Between Intensity Zones (Easy, Moderate, and Hard)

The included studies used different scales to categorize the intensity zones into easy, moderate, and hard. The most used was the Borg CR10 Scale (*n* = 22). However, the Borg Scale 6 to 20 (*n* = 6) and a generic 0-to-10-point scale without images using the Omni verbal cues for adults (OMNI; *n* = 1) were also used. Moreover, different cutoff values were used to classify sessions into easy, moderate, and hard. Considering the Borg CR10 Scale, training sessions were classified as easy (RPE < 3), moderate (RPE 3–5), and hard (RPE > 5) in nine studies [17–19, 25–27, 29, 31, 33]. In the study by Scantlebury et al. [32], the sessions were classified as easy (RPE 1–2), moderate (RPE 3–4), and hard (RPE 5–10). In the study by Figueiredo et al. [15], the training intensity was classified as easy (RPE < 4), moderate (RPE ≥ 4 to ≤ 7), and hard (RPE > 7). Ieno et al. [28] classified the sessions as easy (RPE ≤ 3), moderate (RPE 4–6), and hard (RPE ≥ 7). Besides, a 2-step cluster with log-likelihood as the distance measure and Schwartz’s Bayesian criterion was performed to classify the training load into easy, moderate, and hard sessions based on the Borg CR10 Scale [20]. Using the Borg scale 6 to 20, Brink et al. [5] classified the training sessions as easy (RPE < 13), moderate (RPE 13–14), and hard (RPE > 14). In the study by Voet et al. [34], the sessions were classified as easy (RPE < 11), moderate (RPE 11–14), and hard (RPE > 14). Unlike previous studies, Kraft et al. [30] used the athletes' perceptions to classify the training sessions as easy (RPE ≤ 4), moderate (RPE 5–7), and hard (RPE ≥ 8). In turn, the categorization was based on the verbal anchor descriptors of the scale used (OMNI from 0 to 10 points): RPE (4 = “Somewhat easy,” 6 = “Somewhat hard,” and 8 = “Hard”).

### Study Quality Assessment

The bias scores of the studies included in this systematic review and meta-analysis ranged from four (moderate risk of bias) to six (low risk of bias) out of eight possible points (Table 2). Items 6 and 7 were answered with “No” in all 29 studies following the guidelines of the Quality Assessment Tool of the National Institutes of Health for Observational Cohort and Cross-sectional Studies because they are cross-sectional studies. Items 3, 8, 9, 10, 12, and 13 were considered not applicable to the cross-sectional studies included in this review. Therefore, eight items (1, 2, 4, 5, 6, 7, 11, and 14) were considered to assess the methodological quality of the studies.

**Table 2:** Quality assessment tool for observational cohort and cross-sectional studies

References	Item 1	Item 2	Item 3	Item 4	Item 5	Item 6	Item 7	Item 8	Item 9	Item 10	Item 11	Item 12	Item 13	Item 14	Scores
Andrade et al. [44]	Yes	Yes	NA	Yes	No	No	No	NA	NA	NA	Yes	NA	NA	No	4/8
Andrade Nogueira et al. [25]	Yes	Yes	NA	Yes	Yes	No	No	NA	NA	NA	Yes	NA	NA	No	5/8
Barnes et al. [19]	Yes	Yes	NA	Yes	Yes	No	No	NA	NA	NA	Yes	NA	NA	Yes	6/8
Barroso et al. [26]	Yes	Yes	NA	Yes	Yes	No	No	NA	NA	NA	Yes	NA	NA	No	5/8
Barroso et al. [38]	Yes	Yes	NA	Yes	Yes	No	No	NA	NA	NA	Yes	NA	NA	No	5/8
Brink et al. [5]	Yes	Yes	NA	Yes	Yes	No	No	NA	NA	NA	Yes	NA	NA	No	5/8
Brink et al. [39]	Yes	Yes	NA	Yes	Yes	No	No	NA	NA	NA	Yes	NA	NA	Yes	6/8
Cruz et al. [27]	Yes	Yes	NA	Yes	Yes	No	No	NA	NA	NA	Yes	NA	NA	No	5/8
Doeven et al. [40]	Yes	Yes	NA	Yes	Yes	No	No	NA	NA	NA	Yes	NA	NA	No	5/8
Figueiredo et al. [15]	Yes	Yes	NA	Yes	Yes	No	No	NA	NA	NA	Yes	NA	NA	Yes	6/8
Foster et al. [17]	Yes	Yes	NA	Yes	Yes	No	No	NA	NA	NA	Yes	NA	NA	No	5/8
Ieno et al. [28]	Yes	Yes	NA	Yes	Yes	No	No	NA	NA	NA	Yes	NA	NA	No	5/8
Imamura et al. [41]	Yes	Yes	NA	Yes	Yes	No	No	NA	NA	NA	Yes	NA	NA	No	5/8
Inoue et al. [29]	Yes	Yes	NA	Yes	Yes	No	No	NA	NA	NA	Yes	NA	NA	No	5/8
Kraft et al. [30]	Yes	Yes	NA	Yes	Yes	No	No	NA	NA	NA	Yes	NA	NA	No	5/8
Lupo et al. [45]	Yes	Yes	NA	Yes	Yes	No	No	NA	NA	NA	Yes	NA	NA	Yes	6/8
Medina et al. [42]	Yes	Yes	NA	Yes	No	No	No	NA	NA	NA	Yes	NA	NA	No	4/8
Murphy et al. [49]	Yes	Yes	NA	Yes	Yes	No	No	NA	NA	NA	Yes	NA	NA	Yes	6/8
Nogueira et al. [31]	Yes	Yes	NA	Yes	Yes	No	No	NA	NA	NA	Yes	NA	NA	No	5/8
Oytun et al. [46]	Yes	Yes	NA	Yes	Yes	No	No	NA	NA	NA	Yes	NA	NA	No	5/8
Rabelo et al. [20]	Yes	Yes	NA	Yes	Yes	No	No	NA	NA	NA	Yes	NA	NA	No	5/8
Redkva et al. [16]	Yes	Yes	NA	Yes	Yes	No	No	NA	NA	NA	Yes	NA	NA	No	5/8
Rodrigues-Marroyo et al. [47]	Yes	Yes	NA	Yes	Yes	No	No	NA	NA	NA	Yes	NA	NA	No	5/8
Scantlebury et al. [32]	Yes	Yes	NA	Yes	Yes	No	No	NA	NA	NA	Yes	NA	NA	Yes	6/8
Sinnott-O'Connor et al. [33]	Yes	Yes	NA	Yes	Yes	No	No	NA	NA	NA	Yes	NA	NA	No	5/8
Vaquera et al. [43]	Yes	Yes	NA	Yes	Yes	No	No	NA	NA	NA	Yes	NA	NA	No	5/8
Viveiros et al. [48]	Yes	Yes	NA	Yes	Yes	No	No	NA	NA	NA	Yes	NA	NA	No	5/8
Voet et al. [34]	Yes	Yes	NA	Yes	Yes	No	No	NA	NA	NA	Yes	NA	NA	No	5/8
Wallace et al. [18]	Yes	Yes	NA	Yes	Yes	No	No	NA	NA	NA	Yes	NA	NA	No	5/8

Item 1: Was the research question or objective in this paper clearly stated?; Item 2: Was the study population clearly specified and defined?; Item 3: Was the participation rate of eligible persons at least 50%?; Item 4: Were all the subjects selected or recruited from the same or similar populations (including the same time period)? Were inclusion and exclusion criteria for being in the study prespecified and applied uniformly to all participants?; Item 5: Was a sample size justification, power description, or variance and effect estimates provided?; Item 6: For the analyses in this paper, were the exposure(s) of interest measured prior to the outcome(s) being measured?; Item 7: Was the timeframe sufficient so that one could reasonably expect to see an association between exposure and outcome if it existed?; Item 8: For exposures that can vary in amount or level, did the study examine different levels of the exposure as related to the outcome (e.g., categories of exposure, or exposure measured as continuous variable)?; Item 9: Were the exposure measures (independent variables) clearly defined, valid, reliable, and implemented consistently across all study participants?; Item 10: Was the exposure(s) assessed more than once over time?; Item 11: Were the outcome measures (dependent variables) clearly defined, valid, reliable, and implemented consistently across all study participants?; Item 12: Were the outcome assessors blinded to the exposure status of participants?; Item 13: Was loss to follow-up after baseline 20% or less?; Item 14: Were key potential confounding variables measured and adjusted statistically for their impact on the relationship between exposure(s) and outcome(s)?; NA: Not applicable. Items 6 and 7 were answered with “No” in all 29 studies according to the guidelines of the National Institutes of Health Quality Assessment Tool for Observational Cohort and Cross-Sectional Studies. Items 3, 8, 9, 10, 12, and 13 were considered not applicable to the cross-sectional studies included in this review

Six studies were classified as having a low risk of bias [15, 19, 32, 39, 45, 49], while twenty-three were classified as having a moderate risk of bias [5, 16–18, 20, 25–31, 33, 34, 38, 40–44, 46–48]. All studies in this review included the research question or objective (item 1), clearly specified the study population (item 2), and all subjects were selected or recruited from the same or similar populations (item 4). Twenty-three studies [5, 16–18, 20, 25–31, 33, 34, 38, 40–44, 46–48] did not present key potential confounding variables measured and adjusted statistically (item 14).

### Certainty of Evidence

Using the GRADE approach, the certainty of evidence was very low (Table 3). The downgraded aspects were risk of bias and imprecision, and none of the aspects (effect size, dose–response gradient, or confounding factors) increased the certainty of evidence.

**Table 3:** Approach grading of recommendations assessment, development, and evaluation on the certainty of evidence

Certainty assessment							№ of participants		Effect		Certainty	Importance
No of studies	Study design	Risk of bias	Inconsistency	Indirectness	Imprecision	Other considerations	Coaches	Athletes	Relative (95% CI)	Absolute (95% CI)		
**Overall RPE**												
34	Observational studies	Serious^a^	Not serious	Not serious	Not serious	None	109	602	–	SMD **0.19 SD lower** (0.04 lower to 0.41 higher)	⊕ ◯◯◯ Very low	IMPORTANT
**Overall sRPE**												
21	Observational studies	Serious^a^	Not serious	Not serious	Not serious	None	61	368	–	SMD **0.05 SD higher** (0.24 lower to 0.33 higher)	⊕ ◯◯◯ Very low	IMPORTANT
**Easy RPE**												
20	Observational studies	Serious^a^	Not serious	Not serious	Not serious	None	63	518	–	SMD **0.44 SD lower** (0.87 lower to 0.01 lower)	⊕ ◯◯◯ Very low	IMPORTANT
**Moderate RPE**												
21	Observational studies	Serious^a^	Not serious	Not serious	Not serious	Publication bias strongly suspected^b^	64	536	–	SMD **0.05 SD higher** (0.22 lower to 0.31 higher)	⊕ ◯◯◯ Very low	IMPORTANT
**Hard RPE**												
21	Observational studies	Serious^a^	Serious^c^	Not serious	Not serious	None	64	536	–	SMD **0.41 SD higher** (0.19 lower to 1 higher)	⊕ ◯◯◯ Very low	IMPORTANT
**Easy sRPE**												
8	Observational studies	Serious^a^	Not serious	Not serious	Serious^d^	None	18	114	–	SMD **0.54 SD lower** (1.05 lower to 0.02 lower)	⊕ ◯◯◯ Very low	IMPORTANT
**Moderate sRPE**												
8	Observational studies	Serious^a^	Not serious	Not serious	Serious^d^	None	18	114	–	SMD **0.15 SD lower** (0.66 lower to 0.36 higher)	⊕ ◯◯◯ Very low	IMPORTANT
**Hard sRPE**												
8	Observational studies	Serious^a^	Not serious	Not serious	Serious^d^	None	18	114	–	SMD **0.2 SD higher** (0.3 lower to 0.71 higher)	⊕ ◯◯◯ Very low	IMPORTANT

*CI* confidence interval, *SMD* standardized mean difference

Explanations

a. More than 25% of participants were from studies with a moderate or high risk of bias

b. Egger's linear regression indicated potential publication bias for RPE in the moderate category

c. Heterogeneity *I*^2^ > 50%

d. The total number of participants in this comparison is less than 140

### Meta-Analysis Results

A low heterogeneity (*χ*^2^ = 41.66, *df* = 33, *P* = 0.14; *I*^2^ = 21%) was observed in studies that compared overall RPE between coaches and athletes. Likewise, a low heterogeneity (*χ*^2^ = 3.72, *df* = 20, *P* = 1.00; *I*^2^ = 0%) was observed in studies that compared overall sRPE between coaches and athletes. When comparing the RPE between coaches and athletes in the three effort categories (easy, moderate, and hard), a low heterogeneity was observed for easy category (*τ*^2^ = 0.35; *χ*^2^ = 32.33, *df* = 18, *P* = 0.02; *I*^2^ = 44%), a low heterogeneity for the moderate category (*χ*^2^ = 28.04, *df* = 20, *P* = 0.11; *I*^2^ = 29%), and a moderate heterogeneity for the hard category (*τ*^2^ = 1.19; *χ*^2^ = 68.42, *df* = 19, *P* < 0.00001; *I*^2^ = 72%). When comparing the sRPE between coaches and athletes in the three effort categories, a low heterogeneity was observed for the easy category (*χ*^2^ = 1.18, *df* = 7, *P* = 0.99; *I*^2^ = 0%), a low heterogeneity for the moderate category (*χ*^2^ = 0.69, *df* = 7, *P* = 1.00; *I*^2^ = 0%), and a low heterogeneity for the hard category (*χ*^2^ = 0.65, *df* = 7, *P* = 1.00; *I*^2^ = 0%).

**Fig. 2:**
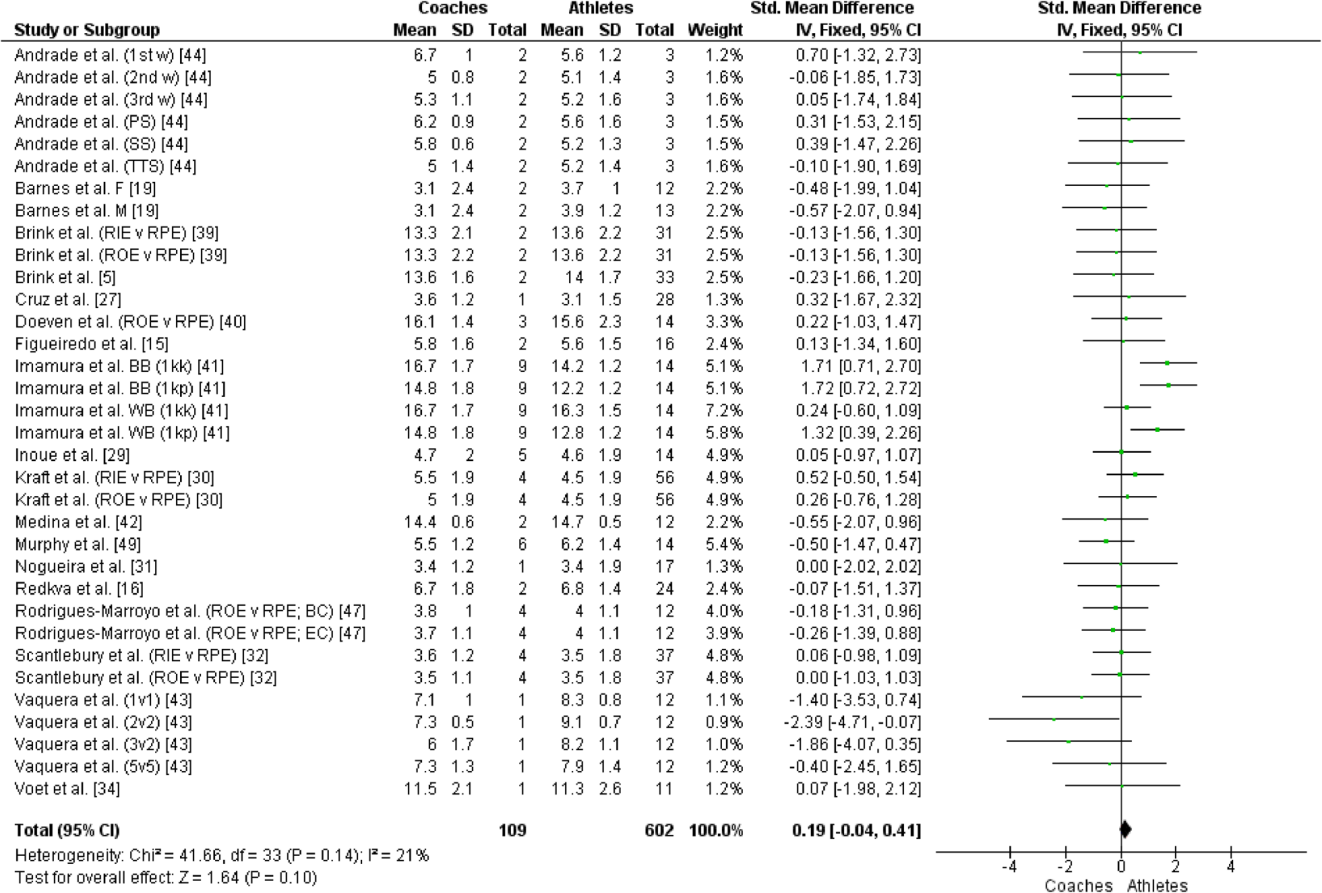
Forest plot comparing overall RPE between coaches and athletes. CI = Confidence interval, IV = Inverse variance, SD = Standard deviation, 1st w = First week, 2nd w = Second week, 3rd w = Third week, PS = Physical sessions, SS = Strength sessions, TTS = Tactical-technical sessions, F = Female, M = Male, v = Versus, RPE = Ratings of perceived exertion, RIE = Ratings of intended exertion by coaches, ROE = Ratings of observed exertion by coaches, BB (1kk) = Black belt (1000 kicks), BB (1kp) = Black belt (1000 punches), WB (1kk) = White belt (1000 kicks), WB (1kp) = White belt (1000 punches), BC = Beginner coaches, EC = Expert coaches, 1v1 = One-a-side game, 2v2 = 2-a-side game, 3v2 = Superiority situations game, 5v5 = 5-a-side game

**Fig. 3:**
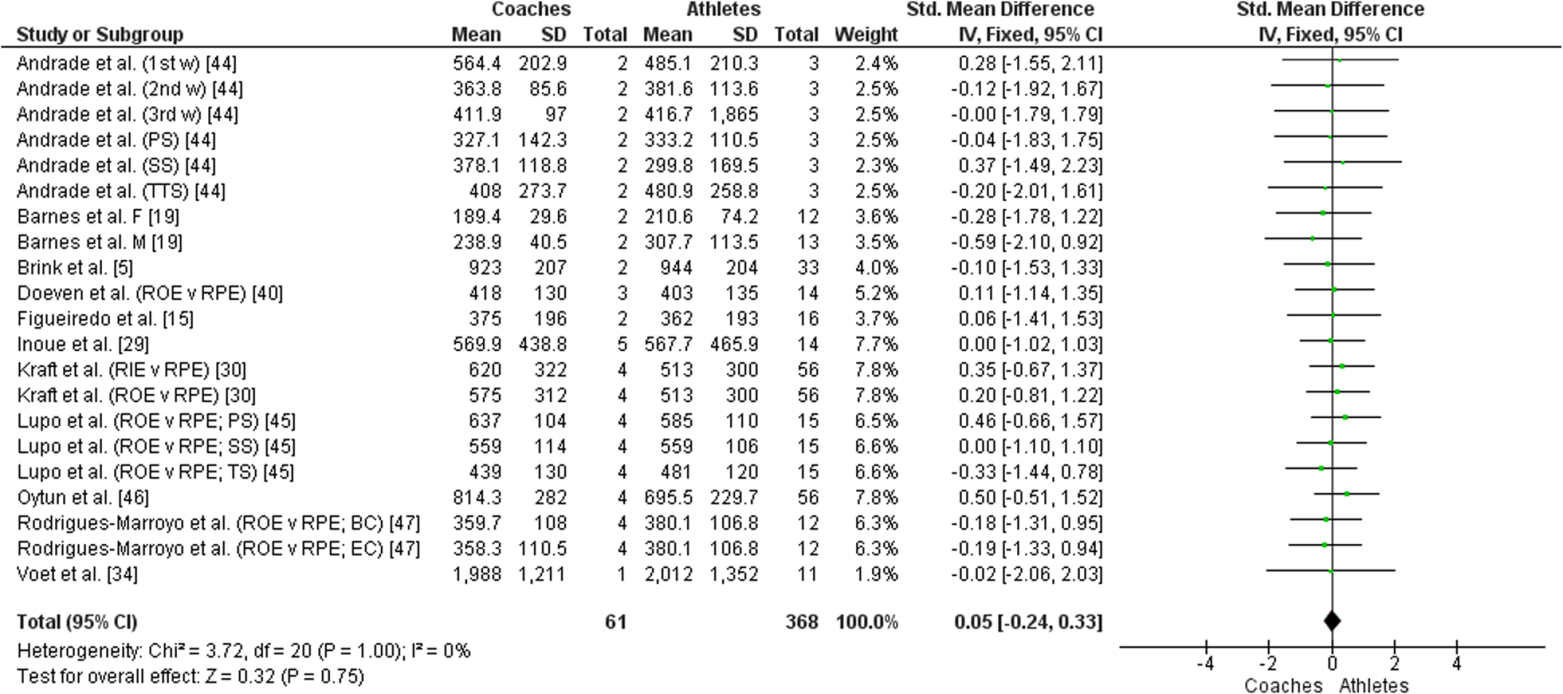
Forest plot comparing overall sRPE between coaches and athletes. CI = Confidence interval, IV = Inverse variance, SD = Standard deviation, 1st w = First week, 2nd w = Second week, 3rd w = Third week, PS = Physical sessions, SS = Strength sessions, TTS = Tactical-technical sessions, TS = Technical sessions, F = Female, M = Male, v = Versus, RPE = Ratings of perceived exertion, RIE = Ratings of intended exertion by coaches, ROE = Ratings of observed exertion by coaches, BC = Beginner coaches, EC = Expert coaches

The overall RPE prescribed/intended/observed by the coaches showed no significant difference when compared to the RPE perceived by the athletes (*Z* = 1.64, *P* = 0.10, SMD = 0.19 [95% CI −0.04 to 0.41]; small effect size, see Fig. 2). Likewise, no significant difference was observed when comparing the overall sRPE prescribed/intended/observed by the coaches and that perceived by the athletes (*Z* = 0.32, *P* = 0.75, SMD = 0.05 [95% CI −0.24 to 0.33]; small effect size, see Fig. 3). This result indicated that the athletes perceived the same intensity and internal load prescribed/intended/observed by the coaches.

**Fig. 4:**
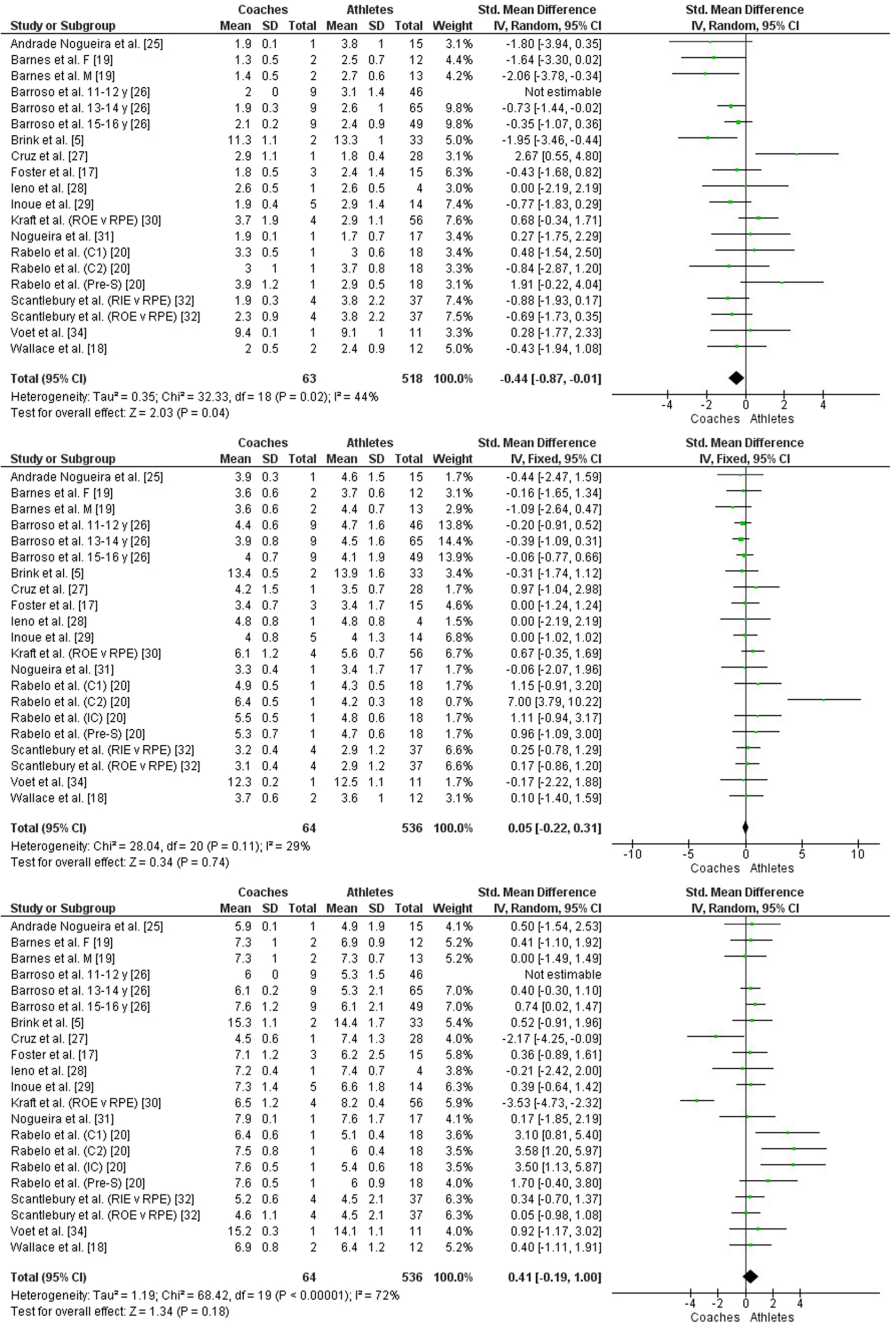
Forest plot comparing RPE between coaches and athletes in three effort categories, easy (top panel), moderate (middle panel) and hard (bottom panel). CI = Confidence interval, IV = Inverse variance, SD = Standard deviation, F = Female, M = Male, y = Years old, v = Versus, RPE = Ratings of perceived exertion, RIE = Ratings of intended exertion by coaches, ROE = Ratings of observed exertion by coaches, Pre-S = Pre-season, C1 = First competitive period, IC = intercompetition period, C2 = Second competitive period

When comparing the RPE prescribed/intended/observed by the coaches and that perceived by the athletes in the easy effort category, a significant difference was observed (*Z* = 2.03, *P* = 0.04, SMD = −0.44 [95% CI −0.87 to −0.01]; small effect size, Fig. 4, top panel). Thus, the athletes perceived an intensity greater than the coaches prescribed/intended/observed. In the moderate effort category, no significant difference was observed when comparing RPE between coaches and athletes (*Z* = 0.34, *P* = 0.74, SMD = 0.05 [95% CI −0.22 to 0.31]; small effect size, see Fig. 4, middle panel. In the hard category, no significant difference was observed between the coaches prescribed/intended/observed and the athletes perceived (*Z* = 1.34, *P* = 0.18, SMD = 0.41 [95% CI −0.19 to 1.00]; small effect size, Fig. 4, bottom panel). This result indicated that the athletes perceived the same intensity prescribed/intended/observed by the coaches.

**Fig. 5:**
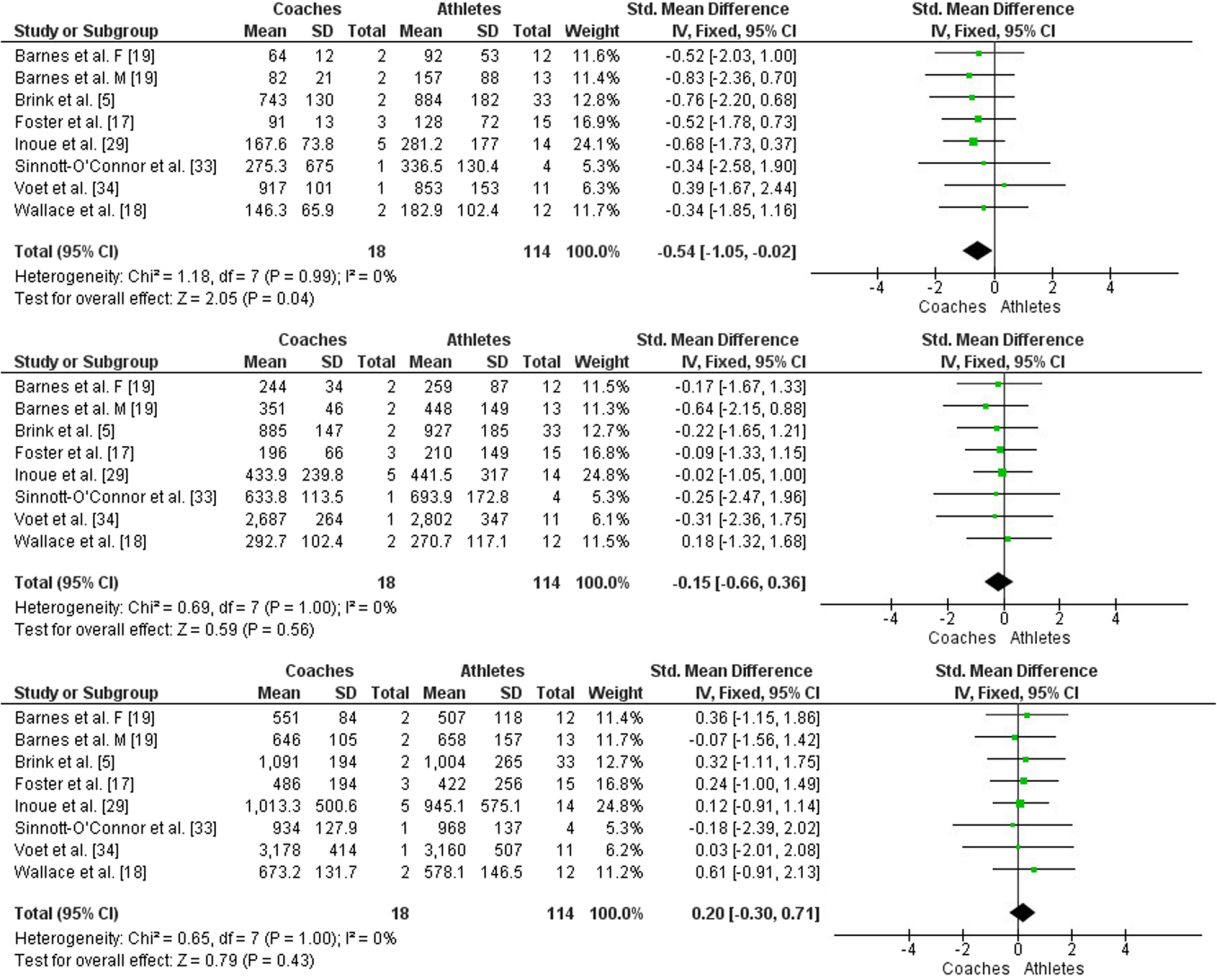
Forest plot comparing sRPE between coaches and athletes in three effort categories, easy (top panel), moderate (middle panel) and hard (bottom panel). CI = Confidence interval, IV = Inverse variance, SD = Standard deviation, F = Female, M = Male

In the comparison between the sRPE prescribed/intended/observed by the coaches and that perceived by the athletes in the easy effort category, a significant difference was observed (*Z* = 2.05, *P* = 0.04, SMD = −0.54, 95% CI [−1.05 to −0.02]; moderate effect size, see Fig. 5, top panel). Thus, the athletes perceived an internal load greater than the coaches prescribed/intended/observed. In the moderate effort category, no significant difference was observed when comparing sRPE between coaches and athletes (*Z* = 0.59, *P* = 0.56, SMD = −0.15 [95% CI −0.66 to 0.36]; small effect size, see Fig. 5, middle panel). In the category of hard effort, no significant difference was observed when comparing the sRPE prescribed/intended/observed by the coaches and that perceived by the athletes (*Z* = 0.79, *P* = 0.43, SMD = 0.20 [95% CI −0.30 to 0.71]; small effect size, see Fig. 5, bottom panel). The results of the moderate and hard effort categories indicate that athletes perceived the same internal load prescribed/intended/observed by the coaches.

Egger's linear regression indicated no potential publication biases for overall RPE (*P* = 0.51), and for RPE in the easy (*P* = 0.37), and hard (*P* = 0.51) categories. However, Egger's linear regression indicated potential publication bias for RPE in the moderate category (*P* = 0.03). No potential publication bias was observed for overall sRPE (*P* = 0.22), and for sRPE in the easy (*P* = 0.10), moderate (*P* = 0.34), and hard (*P* = 0.68) categories, respectively. The funnel plots (Additional file 1: Figure S1 to S3) depict the distribution of these data.

## Discussion

This systematic review and meta-analysis aimed to investigate whether there are differences between the training load perceived by athletes and that prescribed/intended/observed by coaches. The main findings were: (1) No significant differences were observed between the overall RPE and sRPE perceived by the athletes and those prescribed/intended/observed by the coaches (Figs. 2 and 3); (2) Significant differences were observed between the RPE and sRPE perceived by the athletes and those prescribed/intended/observed by the coaches in training sessions classified as easy (Figs. 4 and 5), in which the athletes perceived intensity and internal load greater than that prescribed/intended/observed by the coach; (3) No significant differences were observed between the RPE and sRPE perceived by the athletes and those prescribed/intended/observed by the coaches in training sessions classified as moderate and hard (Figs. 4 and 5).

### Agreement and Disagreement on Training Load Between Athletes and Coaches

The high-performance sport imposes intense training loads on athletes, establishing a complex relationship between an adequate application of these training loads and the recovery process. In this sense, the quantification of training load becomes essential to monitor and prescribe a training program for athletes, thus ensuring increased performance [2]. Therefore, the coaches should know how each athlete perceives the prescribed load of successive training sessions [15]. To accurately prescribe training loads and interpret athlete responses, it is essential to establish an agreement between what was prescribed by the coach and what was perceived by the athlete. This review showed that overall RPE and sRPE were similar between athletes and coaches. This good relationship between coach and athlete is essential to achieve the established goals and optimize individual performance. However, significant differences were observed between the RPE and sRPE perceived by the athletes and those prescribed/intended/observed by the coaches in training sessions classified as easy.

The agreement and disagreement may be related to several factors such as differences in coach supervision during training sessions, physiological and psychosocial factors, communication between coaches and athletes, coach experience, athlete experience [19], competition atmosphere, crowd, motivation, competition outcome, sponsors [40], environmental factors such as temperature and humidity, and factors influencing athlete recovery (diet, sleep, personal stressors) [30].

#### Scale Used

The rationale for differences between the training load perceived by athletes and that prescribed/intended/observed by coaches has not been fully elucidated. However, it seems not to be related to the scale used. There are a variety of scales that can be used to measure RPE. The Borg CR10 Scale, the Borg Scale 6–20, and a generic 0-to-10-point scale without images using the Omni verbal cues for adults (OMNI) were also used in this review. In addition, we observed different cutoff values to classify sessions into easy, moderate, and hard. However, the results seem not to be influenced by scale choice or cutoff values. A good verbal anchorage seems to be important and will allow the athlete to define the intensity zones more precisely [13, 50]. It is essential that athletes understand the scale and accurately link physical sensations to a number on the scale during different exercise intensities [51]. Different questions have been used to measure the RPE and sRPE as, for example, “How hard was your session?”, “How was your training session today?”, “How was your workout?”. A slight difference in the text and the way the question is asked can influence the athlete's perception and response, making comparisons between studies difficult. However, there is no knowledge about the influence of these small changes on the accuracy of the tool's use by coaches [52]. In this sense, these different questions do not seem to influence the results [22, 53]. Thus, there is no ideal scale but the need for familiarization and good verbal anchoring [54]. Besides, Coyne et al. [54] reported that a 100-point RPE category ratio scale (CR100) should be considered to improve sensitivity. The CR100 may have greater sensitivity due to more verbal anchors and a finer grading than the CR10 scale [54]. However, counter-evidence shows interchangeability between the CR100 and CR10 scales [55, 56]. In addition, no study included in this systematic review and meta-analysis used the CR100 scale.

#### Age and Sporting Experience

The agreement/disagreement between coach and athlete can also be influenced by age and sporting experience [15]. Agreement between coaches and athletes tended to increase with age and experience. Based on the results of Barroso et al. [26], it is conceivable that more experienced athletes may perceive effort better than less experienced athletes due to greater variability in stimuli during their years of training. This variability in intensity can improve RPE, allowing athletes to experience and identify various physiological changes, thus creating an internal anchor for their efforts. Another critical point is that the instructions given to younger athletes must be clearly defined, as there is a lack of sports maturity to accurately perceive the intensity of the training load [26]. Misunderstanding instructions can induce athletes to perform tasks at different intensities from those previously planned, affecting RPE. Thus, coaches must be concerned with how to provide information to their athletes. However, more studies are needed to understand better how instruction can affect the relationship between coaches and athletes' RPE. Furthermore, it seems important that young athletes perform training at different intensities to improve their intensity effort perception [26].

#### Observed RPE/sRPE

Brink et al. [39] reported that coaches adjust their perceptions after observing training sessions; however, the incompatibility with players' perceptions remained. In fact, previous findings suggest coaches cannot accurately observe the internal load of players [47, 49]. Scantlebury et al. [32] found that the level of agreement between coach and athlete RPE improved following training with coaches altering their RPE to align with the athlete's. They found the relationship between coach observed and athlete perceived RPE to improve compared to coach intended and athlete perceived RPE. To reduce issues arising from the over/under-prescription of training load, coaches must ensure that desired athlete responses to training are being achieved [32]. Importantly, the coach could re-align the training load if there is a mismatch. The incompatibility can be observed through daily monitoring of recovery and applied training loads. In this sense, coaches should intervene, increasing/decreasing training intensity and/or volume. The underestimation of RPE and sRPE as seen during easy training sessions may predispose the athlete to overuse injury or nonfunctional overreaching through an inability to handle the excess load [32].

#### Endurance Capacity

An individual characteristic influencing RPE is the intermittent endurance capacity [57]. For example, coaches estimate that athletes with a higher intermittent endurance capacity will perceive training to be less intense [39]. It is noteworthy that this characteristic is more evident in team sports. Furthermore, Barroso et al. [38] reported that the greater volume and distance of repetition during interval training influence the classification of the subjective perception of the session, increase the inter-individual variability, and affect the relationship between coaches and athletes. In this sense, care must be taken when prescribing sessions with greater volume and distance.

#### Effort Categories and Female/Male Athletes

There is a tendency to prescribe moderate-intensity training loads [58]. Gearhart et al. [51] proposed that trained athletes can more easily identify the intensity levels they experience most frequently. The discrepancy between athletes and coaches in the easy category may be psychophysiological [6]. There is a trend for athletes to report perceptions of moderate-intensity, which would be the pleasure perception zone. In this sense, low intensity cannot motivate [59, 60]. In addition, since easy or moderate training sessions often follow hard sessions, another explanation could be that coaches may have a misconception of the athlete's physiological state after the training load from the previous session. Although coaches expect an easy training session the day after a hard session, it is possible that athletes do not recover physically or psychologically enough to perceive this training as easy [19]. In an attempt to highlight to coaches which athletes are entering sessions not recovered, quantitative markers could be used to assess recovery (e.g., perceived recovery status scale, total quality of recovery scale, well-being indices, etc.) [56, 61, 62].

There is a lack of data comparing the perceptions of female athletes to coaches in any sport or discipline. In this sense, Barnes' study [19] compared the perceptions of training doses between coaches and male and female cross-country runners. Twenty-five highly-trained cross-country runners (13 male and 12 female) were recruited. The results showed that men and women rated coach-intended easy sessions significantly harder during each month of the season. Men rated moderate intensity sessions significantly higher than coaches, whereas females rated hard intensity sessions significantly lower than coaches. There was no difference between males' and coaches' hard sessions or females' and coaches' moderate sessions. Therefore, men and women report different RPE/sRPE in moderate and hard sessions [19]. The reasons for these discrepancies between male and female athletes are unknown. Studies are controversial when comparing sexes on the perceived exertion scale. Some studies have reported differences in RPE between men and women using different exercise intensity markers (absolute vs. relative) [63, 64]. More recently, Rascon et al. [65] showed no differences in RPE in any of the three exercise intensities (low: < 2 mmol/L, moderate: 2–4 mmol/L, and high: > 4 mmol/L) between men and women.

#### Type of Sport

The coach-athlete mismatch observed in the easy effort category between the studies may be related to the type of sport. It is speculated that for individual sports (cycling, running, swimming), the prescription and monitoring of the training load are easier to perform when compared to team sports. This meta-analysis showed that there is a disagreement between coaches and athletes in the easy effort category regardless of the sport.

#### Coaching Experience

The coach’s experience can also affect the intended/observed training load. In a study included in this systematic review and meta-analyses, the RPE and sRPE of expert (> 10 years) and beginner (≤ 1 year) coaches were compared with the RPE and sRPE of volleyball athletes. The results showed the correspondence between the RPE and sRPE of athletes and coaches, regardless of experience.

#### Training or Match/Games

Good performance in competitions is an important goal of every athlete; however, the proportion of time spent on competitions is small compared to the time spent on training sessions. Doeven et al. [40] showed that the athletes' RPE was lower than ratings of observed exertion by coaches (15.6 ± 2.3 and 16.1 ± 1.4). In this sense, the coach overestimates match exertion. In contrast, Vaquera et al. [43] showed differences between athletes' and coaches' RPE in small-sided games (*P* < 0.002). The coach's RPE was lower when compared to the athletes' RPE. It is noteworthy that during competition trips, coaches may not be aware of the activities performed by athletes in the hours between training sessions. Lack of recovery or additional physical activity can result in accumulated fatigue and greater perceived exertion, even if the external load is similar. Previous research has identified that different intensities and training exercises can influence the mismatch between perceptions of effort [47, 49]. For example, volleyball coaches underestimated players' RPE, particularly during high-intensity fitness exercises. However, the volume of technical-tactical exercises prescribed with moderate intensity corresponded to the dose of exercise received [47]. This result is congruent with data from elite junior tennis athletes, where coaches underestimated the overall RPE of the athletes' training session but not the RPE of the different types of individual exercises [49].

#### Cognitive Demands

Cognitively demanding tasks, such as new tactical concepts in training, can also increase RPE values [39, 66]. This is especially important because cognitive tasks impair physical performance [67]. Besides that, poor education of athletes has been recognized as a limiting factor when using subjective load monitoring procedures. If education around subjective load monitoring is not adequate, athletes may answer dishonestly in an attempt to manipulate future training sessions or to be selected for important competitions [54]. When planning the training, accounting for these issues is a complex and challenging task for coaches.

### Consequences of Divergent Perceptions Between Athletes and Coaches

The effective alternation between training load and recovery theoretically improves sports performance [68]. Signs of inadequate recovery and maladaptation are evident when athletes train more intensely than planned for long periods [4]. In contrast, if athletes do not exert enough effort on the days planned to be intense, training stimuli may not be sufficient to provoke adequate adaptations [17]. The tendency of athletes to report perceptions of moderate training loads can have important implications for training. The tendency of the training load to regress to the mean rather than remain polarized (e.g., easy days and hard days) is considered a common training error [17]. It has been suggested that this decrease in the daily variability of the training load increases monotony [19], a known risk factor for overtraining [22]. Additionally, imprecision in the prescription and training load monitoring are essential factors that increase the risk of injuries and illnesses [22, 69].

For example, Brink et al. [5] reported that soccer athletes perceived the training loads prescribed by the coach to be easy and moderate as harder. At the same time, the athletes perceived the sessions prescribed by the coaches to be hard as easier. The study by Kraft et al. [30] found the opposite, with coaches reporting higher RPE during sessions rated as easy or moderate and lower RPE during sessions rated as hard. This pattern would be preferable because it would indicate greater training variation (i.e., easy and moderate training sessions easier than perceived, while hard sessions were more intense than coaches reported), thus decreasing training monotony and risk of overtraining. It is noteworthy that, unlike published studies, athletes' perceptions were used to classify training sessions as easy, moderate, and hard. Additionally, instead of prescribing RPE before the training session, the coaches in the study by Kraft et al. [30] reported RPE approximately 15–20 min after observing the training session.

Daily control with feedback to coaches is key to decreasing the risk of injury and improving physical performance [69]. In addition, an increased RPE for a typical training session can be used as a guide for coaches to monitor individual increases in fatigue or decreases in fitness levels. On the other hand, a reduction in RPE for these standard training sessions may indicate adaptation to training [18].

### Association Between Coaches and Athletes

A recent systematic review and meta-analysis investigated the relationship between coaches’ rating of intended exertion and/or rating of observed exertion and athletes’ reported rating of perceived exertion (for review, see [52]). A random effect meta-analysis based on 11 studies demonstrated a positive association of athletes' vs. coaches' rating of intended exertion of *r* = 0.62. The pooled correlation from 7 studies of athletes' vs. coaches' rating on observed exertion was *r* = 0.64. In this sense, there was a strong association between coach rating of intended exertion and/or rating of observed exertion and athlete-reported RPE. In our systematic review and meta-analysis, sixteen studies performed a correlation analysis between coaches' and athletes' rating of exertion. Based on the scale of magnitudes proposed by Hopkins (www.sportsci.org): < 0.1, trivial; 0.1–0.3, small; 0.3–0.5, moderate; 0.5–0.7, large; 0.7–0.9, very large; > 0.9, nearly perfect, the results (see Table 1) ranged from small (*r* = 0.24) to nearly perfect (*r* = 0.93) correlation between coaches' and athletes' rating of exertion, thus showing a large variation among studies.

### Strength and Limitations

Some aspects of this review should be highlighted. First, only cross-sectional studies that investigated the differences between the training load perceived by athletes and that prescribed/intended/observed by coaches were included. Although only studies with this design were retrieved in the literature search, we considered this aspect a limitation of this review. This observational characteristic does not determine causality. Second, studies included in this systematic review and meta-analysis were classified as having a low or moderate risk of bias. Future studies should report the main confounding variables to improve internal validity. On the other hand, Egger's linear regression analysis did not indicate potential publication biases that might have significantly influenced the results of overall, easy and hard RPE. However, a potential publication bias was found for RPE in the moderate category. Despite this, no significant difference was observed when comparing RPE between coaches and athletes, with a small effect size. For overall sRPE or in the three effort categories (easy, moderate, and hard), no potential publication biases were found in the present systematic review and meta-analysis. The cutoff values varied from study to study regarding the categorization used (easy, moderate, hard). We consider that familiarizing the instrument and anchoring the descriptors seems to be more important than the cutoff value used; therefore, we believe that the three effort categories did not influence our results. In addition, the 3-category comparison was used in the various studies included in this review [5, 17–20, 25–34]. Third, the certainty of evidence was very low using the GRADE approach, creating a high degree of uncertainty in these results. However, it is noteworthy that observational studies such as those included in this review start with low certainty of evidence. Finally, a limitation of the psychometric scales directly influencing results is the use of artifacts in the scales such as colors, verbal anchors, or figures. Changes in the original scales could influence the athletes' responses and the observed results. Thus, coaches and sports scientists must use the scales initially validated in their original format.

Summarizing this literature is essential to guide coaches' and sport scientists' decision-making in training programming, thus maximizing adaptive responses [6]. Any discrepancies between the program planned by the coach and that executed by the athletes can lead to incorrect prescription/execution of training loads, which are potential causes of the high incidence of negative results in sports training [17].

## Conclusion

Based on the results presented, there is an agreement between coaches and athletes about the overall RPE and sRPE, and RPE and sRPE into moderate and hard effort categories. However, we found divergences between the RPE and sRPE prescribed/intended/observed by coaches and that perceived by the athletes in the easy effort category. Thus, despite a small disagreement, the use of these tools seems to be adequate for training monitoring. However, the certainty of evidence for these results was very low. More studies should be carried out controlling for the risk of bias, imprecision, and confounding factors to increase the certainty of evidence. Researchers, coaches, and athletes must carefully monitor the internal training load, thereby optimizing sports performance, decreasing negative outcomes, and ultimately preventing athletes from developing overtraining.

## Supplementary Information

**Additional file 1**. Search strategy and Funnel plots.

## Data Availability

Data supporting the findings of this study are available from the corresponding author on request.

## Abbreviations

RPE: Rating of perceived exertion
sRPE: Session rating of perceived exertion
RIE: Rating of intended exertion
ROE: Rating of observed exertion
CR10: Category ratio 10 scale
CR100: 100-Point RPE category ratio scale
OMNI: Perceived exertion scale
GPS: Global positioning system
PRISMA: Preferred reporting items for systematic reviews and meta-analyses
GRADE: Grading of recommendations assessment, development and evaluation
SMD: Standardized mean difference
CI: Confidence interval
95% CI: 95% Confidence interval
ES: Effect size
IV: Inverse variance
SD: Standard deviation
mmol/L: Millimoles per liter
NR: Not reported
M: Males
F: Females
Yrs: Years
UCI: Union cycliste internationale
1st w: First week
2nd w: Second week
3rd w: Third week
PS: Physical sessions
SS: Strength sessions
TTS: Tactical-technical sessions
TS: Technical sessions
BB: Black belt
WB: White belt
1kk: 1000 Kicks
1kp: 1000 Punches
BC: Beginner coaches
EC: Expert coaches
1v1: One-a-side game
2v2: 2-a-side game
3v2: Superiority situations game
5v5: 5-a-side game
Pre-S: Pre-season
C1: First competitive period
IC: Intercompetition period
C2: Second competitive period
