# Supplementary material for: Internal Training Load Perceived by Athletes and Planned by Coaches: A Systematic Review and Meta-Analysis
Source: Sports Med Open. 2022 Mar 4;8:35. doi: 10.1186/s40798-022-00420-3 (PMC8897524; doi:10.1186/s40798-022-00420-3)
Supplement: Supplementary file 1 — Additional file 1. Search strategy and Funnel plots. [file 40798_2022_420_MOESM1_ESM.pdf]

Article title: Internal Training Load Perceived by Athletes and Planned by Coaches: A Systematic Review and Meta-Analysis

Journal name: Sports Medicine - Open

Authors: Allan Inoue[1,2,3*], Priscila dos Santos Bunn[2,3], Everton Crivoi do Carmo[4], Eduardo Lattari[5], Elirez Bezerra da Silva[1,2]

[1]Exercise and Sport Sciences Postgraduate Program, Rio de Janeiro State University, Rio de Janeiro, Brazil; [2]Research Group on Exercise and Health Science, Rio de Janeiro State University, Rio de Janeiro, Brazil; [3]Exercise Sciences Research Laboratory, Physical Education Center Admiral Adalberto Nunes (CEFAN), Brazilian Navy, Rio de Janeiro, Brazil; [4]Department of Physical Education, Senac University Center, São Paulo, Brazil; [5]Physical Activity Sciences Postgraduate Program, Salgado de Oliveira University, Niterói, Brazil.

***Correspondence:** Allan Inoue
Brazilian Navy, CEFAN, Exercise Sciences Research Laboratory
Av. Brazil 10590, Rio de Janeiro, RJ, Brazil CEP 21012350.
Tel: +55 22-998891239
E-mail: allan_inoue@hotmail.com

Search strategy:

PubMed

((((((((((((((((SRPE[Title/Abstract]) OR (“Session rating of perceived exertion”[Title/Abstract])) OR (“Session RPE”[Title/Abstract])) OR (“Session-RPE”[Title/Abstract])) OR (“Training Dose”[Title/Abstract])) OR (“Ratings of Perceived Exertion”[Title/Abstract])) OR (“Training load”[Title/Abstract])) OR (“Training Loads”[Title/Abstract])) OR (“Internal load”[Title/Abstract])) OR (“Internal training load”[Title/Abstract])) OR (“External Load”[Title/Abstract])) OR (“External training load”[Title/Abstract])) AND (“Coaches”[Title/Abstract])) OR (“Coach”[Title/Abstract])) OR (“Mismatch Between Coaches-Players Perceptions”[Title/Abstract])) OR (“Coaches-Players Perceptions”[Title/Abstract])) OR (“Discrepancy between coach-athlete perceptions”[Title/Abstract])) OR (“Comparison of Athlete-Coach Perceptions”[Title/Abstract])) OR (“Impaired Player-Coach Perceptions”[Title/Abstract])) OR (“Relationship Between Coach-Athlete Perceptions”[Title/Abstract])

Funnel plots (Figure S1 to S3)

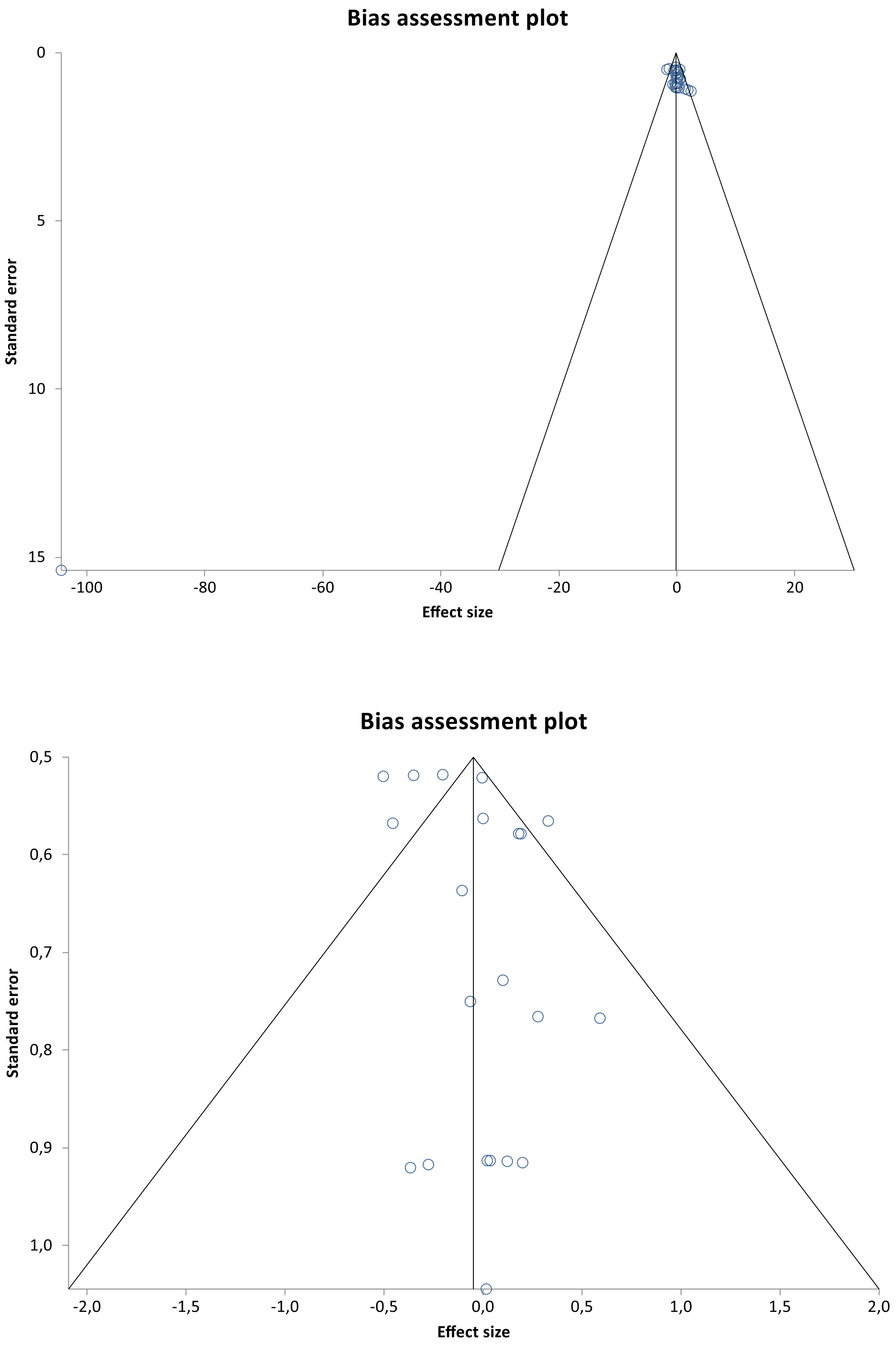

Figure S1. Funnel plots of studies that compared overall RPE (top panel) and overall sRPE (bottom panel) between coaches and athletes.

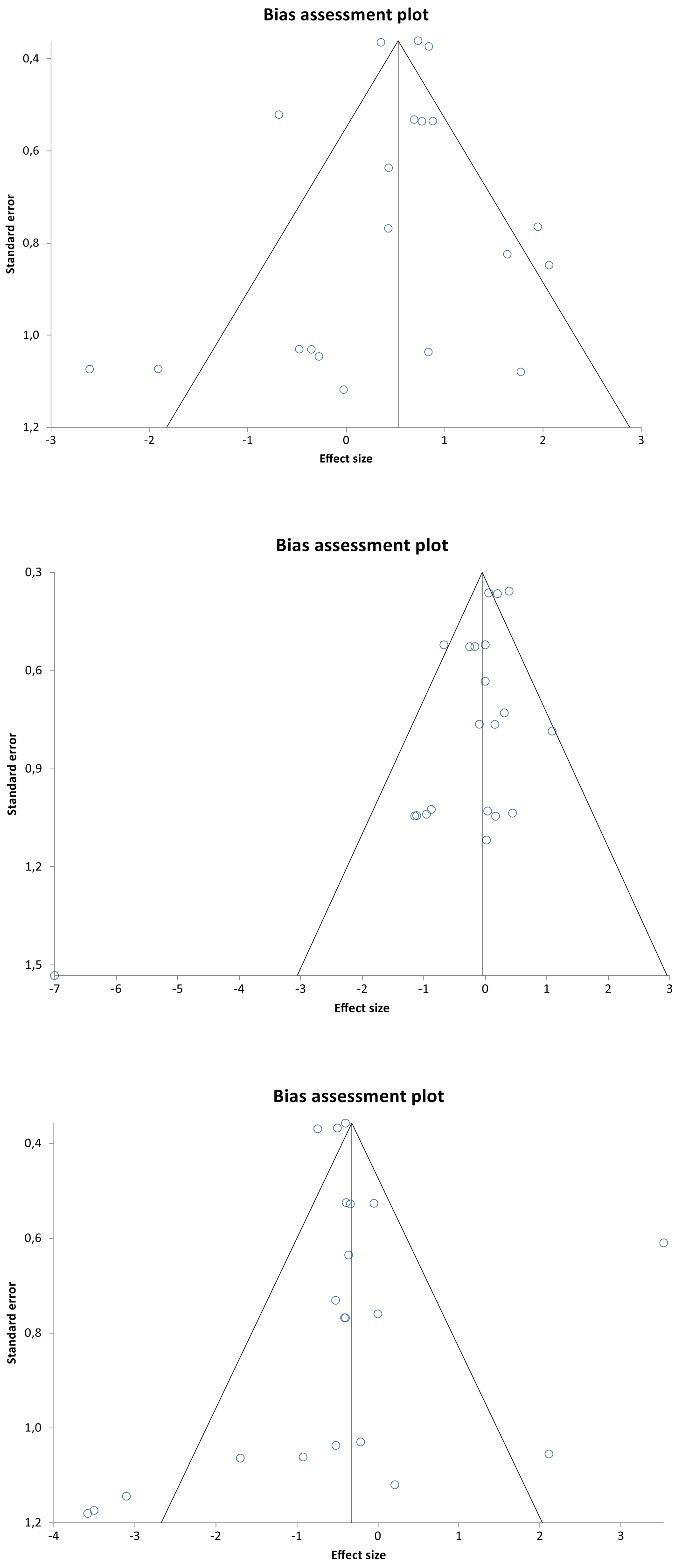

Figure S2. Funnel plots of studies that compared RPE between coaches and athletes in three effort categories, easy (top panel), moderate (middle panel), and hard (bottom panel).

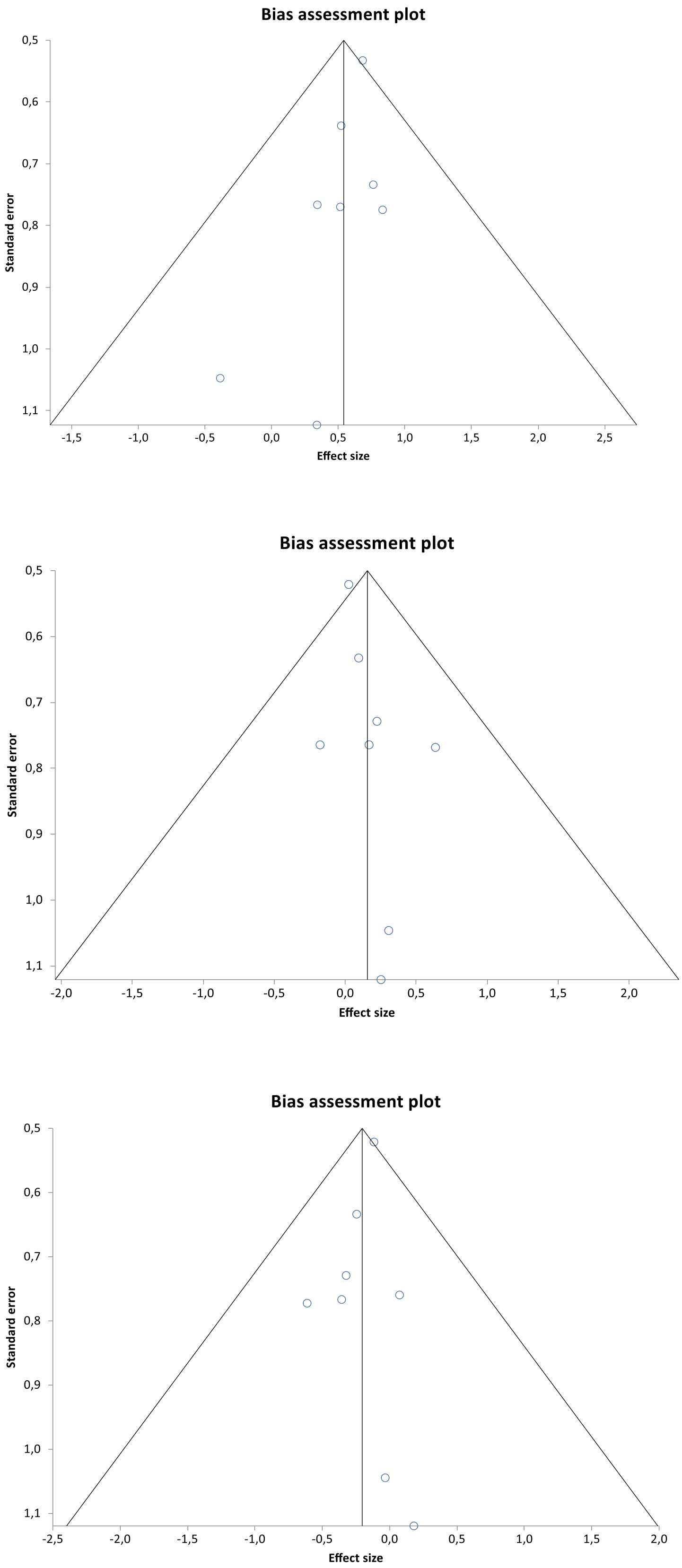

Figure S3. Funnel plots of studies that compared sRPE between coaches and athletes in three effort categories, easy (top panel), moderate (middle panel), and hard (bottom panel).
